# The posterior Cerebellum is involved in constructing Social Action Sequences: An fMRI Study

**DOI:** 10.1038/s41598-019-46962-7

**Published:** 2019-07-31

**Authors:** Elien Heleven, Kim van Dun, Frank Van Overwalle

**Affiliations:** 1 Vrije Universiteit Brussel, Brussels, Belgium; 2 Universiteit Hasselt, Hasselt, Belgium

**Keywords:** Social neuroscience, Human behaviour

## Abstract

Social neuroscience largely ignored the role of the cerebellum, despite its implications in a broad range of tasks and neurological disorders related to social functioning and inferences on others’ mental state such as beliefs. One hypothesis states that during human evolution, the cerebellum’s function evolved from a mere coordinator of fluent sequences of motions and actions, to an interpreter of action sequences without overt movements that are important for social understanding. The present study introduces new tasks to investigate the role of the cerebellum in sequencing, in which participants generated the correct chronological order of new or well-known event stories with or without social elements during functional neuroimaging (fMRI). Results showed strong cerebellar activation during order generation for all event types compared to passive viewing or reading events. More importantly, new social events involving true or false beliefs showed stronger activation in the bilateral posterior cerebellum (Crus 1 and Crus 2) compared to routine social and non-social (mechanical) events. This confirms the critical role of the posterior cerebellum in the understanding and construction of the correct order of action sequences relevant for social understanding. The present tasks and results may facilitate diagnoses and treatments of cerebellar dysfunctions in the future.

## Introduction

Researchers have made great progress in uncovering the neural correlates of social understanding. This advancement, however, was predominantly focused on the cerebrum and the cortical areas subserving mentalizing, collectively termed the mentalizing network (for reviews see^1–3^). This “Mentalizing” or “mind reading” capacity refers to the inference of other persons’ mental states based on their actions, such as their intentions and beliefs, and is crucial to engage in and understand social behavior.

Neuroscientists largely ignored the role of the cerebellum in social reasoning, although the cerebellum is implicated in a broad range of neuropsychiatric and neurodevelopmental disorders related to social functioning such as autistic spectrum disorders (ASD), attentional deficit and hyperkinetic disorder (ADHD), depression, and schizophrenia^4–7^. Nevertheless, recent neuroscientific research made it increasingly clear that the cerebellum plays a more critical role in social thinking than assumed so far. In a large-scale meta-analysis on social cognition and the cerebellum that included functional magnetic resonance imaging (fMRI) studies with healthy humans, Van Overwalle and colleagues found robust activation of the cerebellum during social judgments, including judgments involving mentalizing^8^. There is also strong neural connectivity between the cerebellum and cerebrum during social mentalizing, as revealed by a recent meta-analytic connectivity study on social cognition^9^ and a functional connectivity study pooled across fMRI studies^10^. In addition, based on resting-state connectivity, Buckner and colleagues^11^ identified a distinct mentalizing network in the posterior cerebellum that was directly connected to the mentalizing network in the cerebrum. Both mentalizing networks are part of a larger default mode network^12–14^. Together, these studies documented that the posterior cerebellum (most often Crus 1 and 2) is strongly implicated in social mentalizing, and connected to cortical areas known to be involved in social cognition^2,3^.

What is the mechanism through which the cerebellum exerts its influence on social mentalizing? Given its classical role in motor production, a number of authors have argued that the primary function of the cerebellum is to support sequence learning and recollection of frequently used sequences that underpin skill and acquisition of fluent motion, which develops slowly with practice and is inaccessible to consciousness^15–18^. To do this, the cerebellum constructs internal models of motor processes involving sequencing and planning of movement and compares these with feedback from external and proprioceptive sources, in order to automatize and fine-tune voluntary motor processes and to allow adjustment of movement during its execution. According to these authors, during human evolution, a more advanced function developed which allowed the cerebellum to construct internal models of purely mental processes in which event sequences play a role, without overt movements and somatosensory feedback. In sum, “the cerebellum detects and simulates repetitive patterns of temporally or spatially structured events and generates internal models that can be used to make predictions” (^18^; p.35).

Earlier clinical studies with cerebellar patients have addressed dysfunctions of social cognition without a focus on the sequencing of actions, and reported mixed findings in comparison with healthy controls. Sokolovsky *et al*.^19^ reported impairments in some but not all cerebellar patients, while Roca and colleagues^20^ found no significant differences in mentalizing (Faux Pas test)^21^. In addition, Hoche *et al*. found worse performance on the Reading the Mind in the Eyes test^22^.

In a first study investigating action sequencing, Leggio *et al*.^23^ presented cartoon-like drawings and verbal sentences which had to be reproduced in the chronologically correct sequence. They found that cerebellar patients performed worse on both sequencing tasks than healthy matched controls. Using a very similar task with action photos, Cattaneo and colleagues^24^ reported that cerebellar patients performed significantly worse, especially on photos of biological action and less so on mechanical movements. These latter two studies on action sequencing clearly point to the potential diagnostic value of tasks in which an adequate chronological order of actions has to be generated. However, participants did not engage in inferences on others’ beliefs, so that the implications on social mentalizing are unclear. Therefore, based on these studies we cannot determine whether sequence generation during simple action observation is the only key deficit in cerebellar patients, or whether more advanced social processes also play a role, such as mentalizing processes in which inferences of others’ beliefs have to be made.

**Figure 1:**
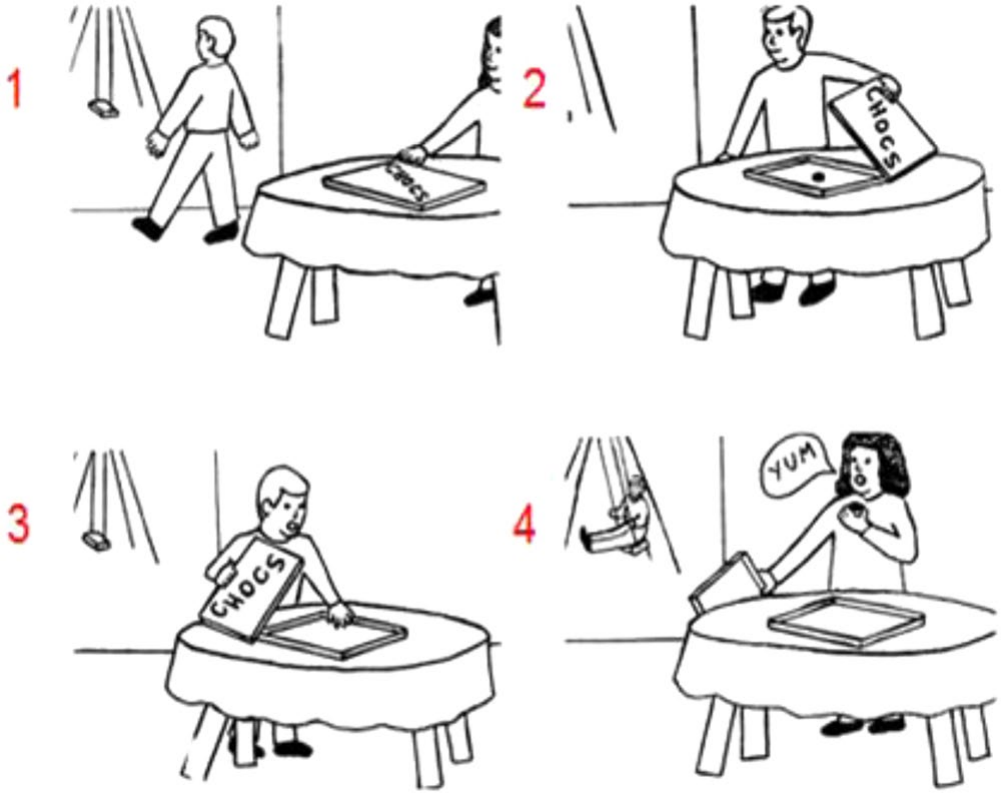
An example of a false belief sequence in the Picture Sequencing task (Langdon & Coltheart^26^; the correct order is 2 – 1 – 4 – 3; the numbers are not shown to the participants but given here for display purposes). Participants had to select, in the correct order, the first picture on the screen, then the second picture, and so on. Each time, the pictures moved in the order indicated by the participant.

In order to explore this possibility, a recent pilot study with cerebellar patients enforced inferences on others’ mental beliefs. The task involved stories in which an agent holds a false belief, that is, beliefs that differ from reality and result in particular behaviors. For instance, an agent looks for an object in the wrong location due to a false belief on the object’s location as a result of a displacement during the agent’s absence^25^. Consequently, participants have to infer the agent’s false belief in order to understand the agent’s behavior. False belief tasks are a crucial test of the capacity to mentalize, because they require the understanding that another person may hold mental beliefs that are contradicted by reality and our own beliefs. Van Overwalle *et al*.^25^ used the *picture sequencing* task with cartoon-like drawings developed by Langdon and Coltheart (^26^ see Fig. 1) which was inspired by an earlier version of this task^27^. The results showed clear impairments of cerebellar patients compared to healthy matched controls on false belief story sequences which involved novel (i.e. non-routine) action sequences, and (close to) normal performance on routine actions (i.e. social scripts) and non-social mechanical movement sequences. This is in line with the hypothesized role of the cerebellum in interpreting novel action sequences during social mentalizing. However, these findings cannot exclude the possibility that this impairment is due to action novelty rather than false belief understanding.

The present study further explores the role of the cerebellum in social mentalizing, focusing on healthy participants. We adapted and extended the earlier patient study by Van Overwalle and colleagues^25^ by addressing two limitations. First, this prior study was limited to false beliefs. The addition of a true belief condition will allow us to disentangle the involvement of the cerebellum for understanding novel events or beliefs in general as opposed to false beliefs. True belief stories require some minimal mental state inference and are relatively novel, so that we might expect significant cerebellar activation also during true belief stories compared to routine actions. However, if false belief understanding is key for cerebellum involvement, we expect true belief stories to engage the cerebellum to a lesser extent than false beliefs. Second, we developed a parallel verbal *story sequencing* task based on the Faux Pas test by Baron-Cohen *et al*.^21^, involving the same conditions of true and false beliefs, and routine actions and mechanical events. This extends the picture task, allows exploration of potential differences in input modality (visual versus verbal) and provides more stimulus material.

Our hypothesis is that ordering action sequences, especially for novel actions involving false (and perhaps also true) beliefs depends on the activation of specific regions in the posterior cerebellum (i.e., Crus 1 and 2) in comparison with overlearned social and non-social sequences. Support for this hypothesis will convincingly demonstrate that the cerebellum has an important function in social cognition.

## Method

### Participants

For the Story Sequencing task, participants were 24 right handed, native Dutch speaking individuals (9 men) with ages varying from 19 to 34 years (M = 22.38 y; SD = 3.00 y); for the Picture Sequencing task, participants were 49 right handed, native Dutch speaking individuals (24 men) with ages varying from 19 to 36 years (M = 23.35 y; SD = 4.26 y). Since we had only 4 trials per condition for the Picture Sequencing task, we invited more participants for this task in order to increase statistical power to at least the same degree as in the experiment of Langdon and Coltheart^26^ in which 40 participants ordered 4 trials per condition. There were 24 participants who performed both tasks in a counterbalanced order. Another 25 participants performed only the Picture Sequencing task together with another task in a counterbalanced order. This other task involved predictions based on trait and causal attributions, did not activate the cerebellum, and is therefore not reported here. All participants were university students that reported no abnormal neurological history and had normal or corrected-to-normal vision. Informed consent was obtained in a manner approved by the Medical Ethics Committee at the Hospital of University of Ghent, where the study was conducted. The experimental protocol was approved by the University of Ghent and performed in accordance with relevant guidelines and regulations. Participants were paid 20 euro in exchange for their participation.

### Sequencing Tasks

#### Picture sequencing

Participants saw 16 cartoon-like scenarios that represented 4 mechanical, 4 social script and 4 false belief events (for an example see Fig. 1) from the original task developed by Langdon and Coltheart^26^. In addition, they saw 4 true belief events that were created by taking the false belief events and changing minimally the order of the event and role of the agents, so that all false beliefs were removed. Since most elements of the events were identical, these true stories were strictly matched counterparts of the false belief stories. Picture sequences were pilot tested on sequencing accuracy on 39 additional participants that did not participate in the fMRI experiment. Given the high accuracy, all the stories were retained (all stimuli are available upon request). For two false belief stories and for the last 8 participants, a few small background details were slightly altered without influencing the general interpretation of the events to improve accuracy inside the scanner (i.e., from the first 41 participants to the last 8 participants, accuracy increased from 4.39 to 5.38 for one story and from 4.95 to 5.00 for another story, with a maximum accuracy of 6; see scoring details in the stimulus material section). Although a better choice would have been to keep all pictures identical during the experiment, we included this last set of 8 participants (with a few slightly altered pictures) in the analysis because the implied process is essentially identical, and lead to very similar behavioral and fMRI results (except the accuracy rates). As a control condition, we added a non-sequential task, which involved simply viewing 4 pictures in their normal order and answering a factual question about its content (all stimuli are available upon request).

#### Story sequencing

From the Faux Pas test^21^, we developed 9 false belief and 9 true belief sequential events. The Faux Pas test^21^ consists of 20 short stories of about equal length that can easily be broken up in 4 sentences. These sentences were reformulated in order (1) to obtain pure false or true belief stories (extracted from the 10 experimental and 10 control stories respectively) because some stories involved Faux Pas or otherwise socially inappropriate behaviors rather than false beliefs, and (2) to make sure that the chronologically correct sequence could be adequately inferred, by adding time constraints (e.g., “Next…”, “After…” etc.). Additionally, we created 9 mechanical and 9 social script sequential events which were in part inspired by the Picture sequencing task^26^ and in part developed from scratch. We created 10 events for each condition. We pilot tested these sequences on 20 additional participants that did not participate in the fMRI experiment, and retained 9 events per condition based on the overall accuracy (the full test is available in the Supplementary Material). For the fMRI study we added a passive reading control condition consisting of 9 trials, similar to picture sequences. The entire task can be found in the Supplementary Material in Dutch and English, as well as all experimental trials in French and Italian.

#### Autism Questionnaire

In order to explore the effect of individual differences of ASD on cerebellar activation as suggested by previous research^4–7^, participants filled out the Dutch version of the Autism Questionnaire (AQ)^28^, originally developed by Baron-Cohen, and colleagues^29^. A score above 32 indicates ASD symptoms.

### Procedure

The two tasks were presented in a counterbalanced order across participants and followed the same procedure. The Picture and Story Sequencing tasks started each with the control condition. Instructions of the control condition were shown on the screen, then a practice trial preceded the actual control trials. Each trial started with a fixation cross (1 s), followed by the presentation of 4 pictures/sentences in their correct chronological order with a factual question at the bottom of the screen. Participants gave a self-paced response to the questions.

Second, the main experimental task was presented. Instructions were given followed by 2 practice trials and all experimental trials. Each trial started with a fixation cross (1 s), followed by the presentation of 4 pictures/sentences in a random order, and participants had to line up the pictures/sentences in the correct order at a self-paced tempo. They had to indicate the correct order by first selecting the first picture/sentence on the screen, then the second picture/sentence, and so on. Each time, the pictures/sentences moved on the screen along the order indicated. At the end of each trial, participants could cancel and redo the trial to correct possible mistakes, or end the trial.

Before entering the scanner, participants were shown 2 practice trials and were familiarized with the option to cancel and redo a trial to correct possible mistakes. We instructed participants to keep on looking for the correct order in the story elements, even if they realized they had made a mistake. We informed them that when they cancelled a trial, all story elements stayed on the screen as they had chosen them so far. Putting as many elements in the correct order thus had its benefits and they could redo a trial relatively quick. We gave these instructions to make sure that processes related to sequence detection and generation would proceed uninterrupted at the beginning of the trials. All trials were presented in a random order. After scanning, participants completed the AQ.

### Imaging procedure

Images were collected with a 3 Tesla Siemens Magnetom Prisma fit scanner system (Siemens Medical Systems, Erlangen, Germany) using a 64-channel radiofrequency head coil. Stimuli were projected onto a screen at the end of the magnet bore that participants viewed by way of a mirror mounted on the head coil. Stimulus presentation was controlled by E-Prime 2.0 (www.pstnet.com/eprime; Psychology Software Tools) running under Windows XP. Participants were placed head first and supine in the scanner bore and were instructed not to move their heads to avoid motion artifacts. Foam cushions were placed within the head coil to minimize head movements. First, a high-resolution anatomical images were acquired using a T1-weighted 3D MPRAGE sequence [TR = 2250 ms, TE = 4.18 ms, TI = 900 ms, acquisition matrix = 256 × 256 × 176, sagittal FOV = 256 mm, flip angle = 9°, voxel size = 1 × 1 × 1 mm]. Second, a fieldmap was calculated to correct for inhomogeneities in the magnetic field^30^. Next, whole brain functional images were collected in a single run using a T2*-weighted gradient echo sequence, sensitive to BOLD contrast (TR = 2000 ms, TE = 29 ms, image matrix = 64 × 64, FOV = 224 mm, flip angle = 90°, slice thickness = 4.0 mm, distance factor = 10%, voxel size = 3.5 × 3.5 × 4.0 mm, 35 axial slices, acceleration factor GRAPPA = 2).

### Image processing

SPM12 (Wellcome Department of Cognitive Neurology, London, UK) was used to process and analyze the fMRI data. To remove sources of noise and artifact, data were preprocessed. Inhomogeneities in the magnetic field were corrected using the fieldmap^30^. Functional data were corrected for differences in acquisition time between slices for each whole-brain volume, realigned to correct for head movement, and co-registered with each participant’s anatomical data. Then, the functional data were transformed into a standard anatomical space (2 mm isotropic voxels) based on the ICBM152 brain template (Montreal Neurological Institute), which approximates Talairach and Tournoux atlas space. Normalized data were then spatially smoothed (6 mm full-width at half-maximum, FWHM) using a Gaussian Kernel. Finally, using the Artifact Detection Tool software package (ART; http://web.mit.edu/swg/art/art.pdf; http://www.nitrc.org/projects/artifact_detect), the preprocessed data were examined for excessive motion artifacts and for correlations between motion and experimental design, and between global mean signal and experimental design. Outliers were identified in the temporal differences series by assessing between-scan differences (Z-threshold: 3.0 mm, scan to scan movement threshold: 0.5 mm; rotation threshold: 0.02 radians). These outliers were omitted from the analysis by including a single regressor for each outlier. Six directions of motion parameters from the realignment step as well as outlier time points (defined by ART) were included as nuisance regressors. A default high-pass filter was used of 128 s and serial correlations were accounted for by the default auto-regressive AR(1) model.

### Behavioral analysis

For each in-scanner task we calculated the mean accuracy, based on a 6-point score for each trial analogous to the scoring system of the original Picture Sequencing task^26^. Two points were given for each first or last picture/sentence if it was placed correctly, and one point for each middle picture/sentence if it was placed correctly. For the response times of the in-scanner task, a distinction was made between (1) the time needed from the random presentation of the stimuli to select the first picture/sentence (first RT) taking all trials into account, and (2) the time needed to find and complete the ordering (total RT), excluding cancelled trials. The percentages of cancelled trials were 10.5% of the story trials, ranging from 9.2% to 12.5% per condition, and 13.1% of the picture trials, ranging from 7.7% to 20.4% per condition. This corresponds to a mean of 1 trial or less per condition over all participants. Participants never cancelled more than once on a trial.

Repeated measures ANOVA was used to compare the accuracy and RTs with condition as the within-subject factor. A Greenhouse-Geisser correction was used if sphericity was not assumed. Partial eta squared was calculated as a measure of effect size. Paired samples t-tests, uncorrected, revealed the pairwise differences when the ANOVA indicated significant differences between groups.

### fMRI analysis

The general linear model of SPM12 (Wellcome Department of Cognitive Neurology, London, UK) was used to conduct the analyses of the fMRI data. At the first (single participant) level, for each task, the event-related design was modeled for each condition (i.e. non-sequential control, mechanical scripts, social scripts, true belief, and false belief) time-locked at the presentation of the pictures/sentences, and convolved with a canonical hemodynamic response function with time and dispersion derivatives. Event duration was set to 4 sec for all conditions of the picture task, and set to 5 sec for the story task, as this was approximately the minimal time needed for most participants to view the pictures/read the sentences and to provide a first quick response (i.e. until they selected the first picture/sentence on the screen). We suspected that the first seconds of each task would be most critical in the process of solving the correct sequence, while additional time probably involved processes related to remembering and updating the found sequence. However, because we had no strong *a priori* notions of the most essential time period, we performed additional analyses involving other time periods. Specifically, we also performed analyses with event duration set to the time needed to provide the first response on each trial individually. These resulted in similar activations but with less statistical significance (in comparisons between experimental conditions) and are therefore not reported here. We also tested other onsets (5 seconds before the first response) and durations (the whole time till the final response; excluding cancellations with re-attempts because we suspected other processes to be involved when participants know to be wrong and try again), but these generally provided weaker activations (probably due to confounds of various processes involving sequencing, recollection and updating) and are not reported. Given that the beginning period of each trial was most essential for the analysis, we included all trials regardless of whether they were answered correctly or not, or were cancelled or not, since this early process is not influenced by the correctness or a cancellation of the trial.

At the second (group) level, we first conducted a whole-brain random effects analysis using a within-participants one-way analysis of variance, contrasting each experimental condition with the non-sequential control condition. Clusters were considered significant at a whole brain Family Wise Error (FWE) corrected peak threshold p < 0.05, with a minimum cluster extent of 10 voxels. Next, we conducted comparison between the false and true belief conditions, in comparison with the other routine (social script or non-social mechanical) experimental conditions, as well as between the true and false belief conditions. In addition, we conducted comparisons between the routine social script and non-social mechanical conditions, as well as all remaining reverse contrasts. We used the same whole-brain threshold for the stories task. However, given that the picture task showed little results (with is to be expected given the lower number of trails and hence less precise estimates), we lowered the threshold to an uncorrected cluster-defining threshold of p < 0.001, followed by a cluster-wise FWE corrected threshold p < 0.05, with a minimum cluster extent of 10 voxels.

In addition, we performed two parametric regression analyses involving participants’ mean accuracy or AQ scores as covariates, to test whether this would reveal activation in the cerebellum. The latter analysis with AQ scores was preformed to explore a potential relationship between autistic characteristics and cerebellum activation.

## Results

### Behavioral results

Mean accuracy, total and first RT (for correct trials only) are listed in Table 1, as well as direct comparisons between conditions by paired t-tests. Taken together, these measures confirm that the false beliefs (and for the story task, also true beliefs) are more difficult as they result in less accuracy and longer RTs.

**Table 1:** Means, Standard deviations and Paired t-tests for the Story and Picture Sequencing tasks.

	Means (Standard deviations)			
	False Belief	True Belief	Social Script	Mechanical
**Story Task** (n = 24)				
Accuracy	5.8_cb_ (0.3)	5.7_c_ (0.4)	6.0_a_ (0.1)	5.9_ab_ (0.2)
RT total (sec)	28.9_c_ (5.9)	27.7_c_ (6.0)	21.3_a_ (4.4)	23.0_b_ (5.2)
RT first (sec)	16.9_c_ (4.0)	15.8_b_ (4.0)	12.9_a_ (3.2)	13.1_a_ (3.5)
**Picture Task** (n = 49)				
Accuracy	5.1_c_ (0.8)	5.7_a_ (0.5)	5.8_a_ (0.4)	5.8_a_ (0.6)
RT total (sec)	22.6_c_ (5.4)	20.7_b_ (4.7)	17.5_a_ (3.8)	19.7_b_ (4.9)
RT first (sec)	13.5_c_ (3.6)	11.5_b_ (2.7)	9.9_a_ (2.4)	11.8_b_ (3.6)

Note: Standard deviations between parentheses. Accuracy totals 6 points for each trial and is determined by 2 points for a correct first and last position, and 1 point for the other intermediate positions. RT total = response time on the total trial not including the cancelling of the initial response; RT first = response time of the first response. Values per row that share the same subscript (a, b, or c) do not differ significantly on a paired t-test with *p* < 0.05, uncorrected.

#### Story sequencing

A repeated measures ANOVA revealed a significant difference in accuracy between conditions (F(3, 69) = 6.46, p = 0.001, η^2^ = 0.22), mainly due to the lower accuracy of the true and false belief conditions compared to social scripts and mechanical events which were almost all significant (for specific differences between conditions, see Table 1). For total RTs, a significant difference was found by the repeated measures ANOVA (F(3, 69) = 39.47, p < 0.001, η^2^ = 0.63), and was due to the slower total RTs between the (false and true) beliefs and the other conditions (social and mechanical scripts). These differences were also significant when comparing the first RTs (F(3, 69) = 26.58, p < 0.001, η^2^ = 0.54).

#### Picture sequencing

A repeated measures ANOVA indicated a significant difference in accuracy between conditions (F(2.51, 120.82) = 18.61, p < 0.001, η^2^ = 0.28). This was due to a significant lower accuracy for the false belief condition compared to all other conditions (for specific differences between conditions, see Table 1). The same ANOVA revealed a significant difference in total RTs (F(2.58, 123,98) = 17.63, p < 0.001, η^2^ = 0.27), which was related to a slower total RT for false beliefs compared to social scripts. The ANOVA also showed differences for the first RTs (F(2.54, 121.69) = 17.07, p < 0.001, η^2^ = 0.26), revealing a slower first RT in the comparison of the same conditions.

#### AQ

All but one participant completed the AQ. Mean score was 16.71, with a range between 3 and 30, well below the clinical threshold of 32.

### fMRI results

**Figure 2:**
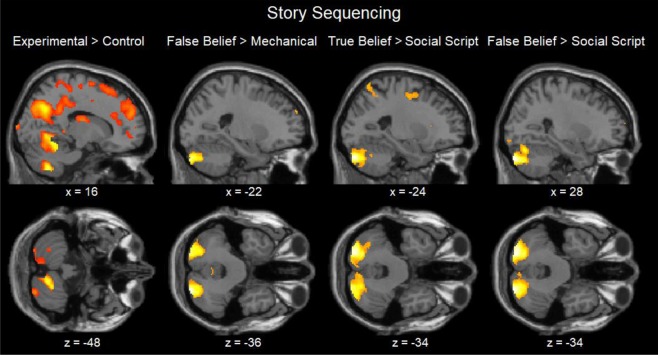
Sagittal and Transverse views of the contrasts during the Story Sequencing task at an uncorrected threshold of *p* < 0.001.

**Figure 3:**
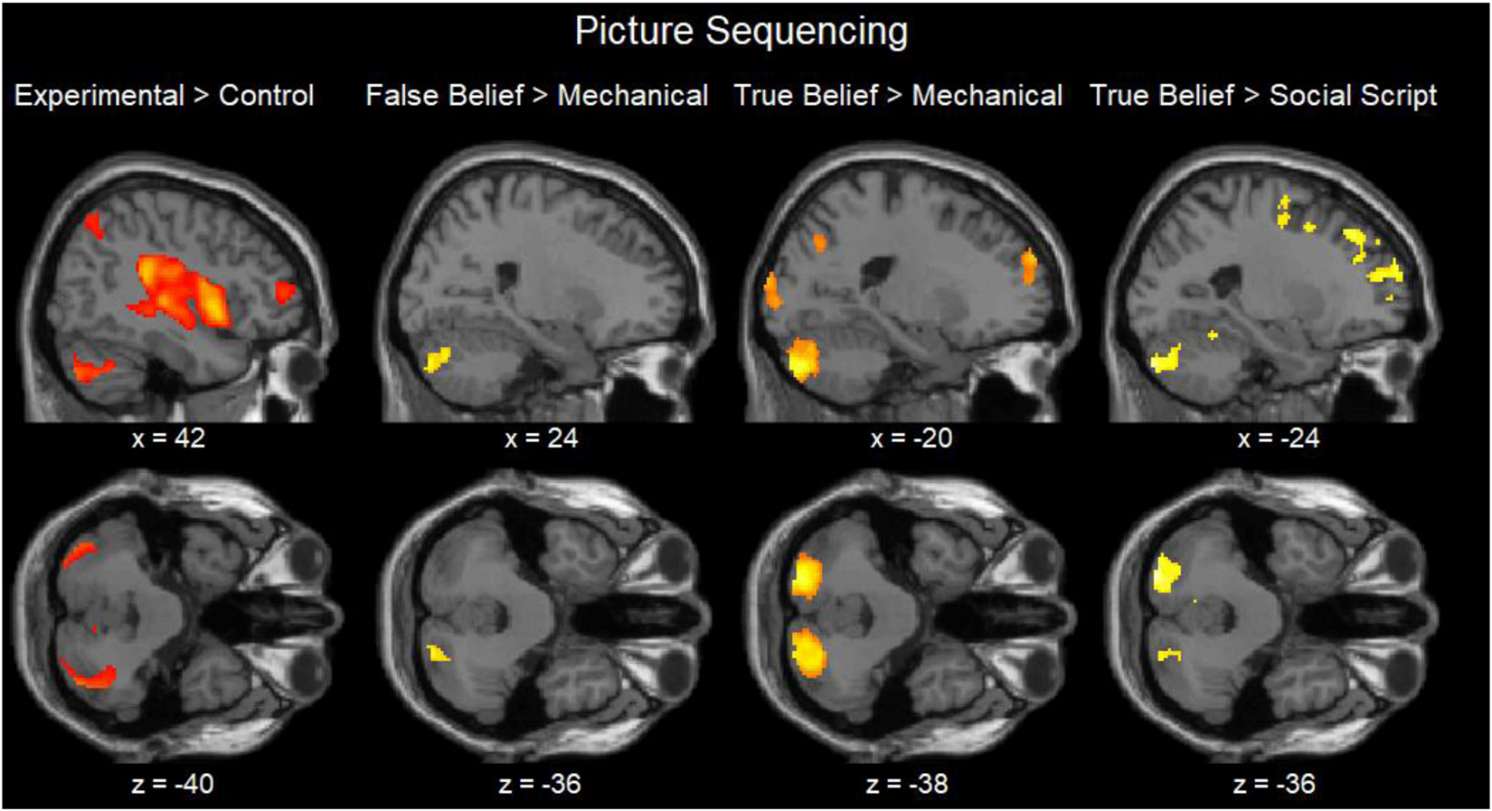
Sagittal and Transverse views of the contrasts during the Picture Sequencing task at an uncorrected threshold of *p* < 0.001.

In order to test whether the cerebellum is involved in social and non-social sequencing of actions and events, we first tested differences between each of the experimental conditions in comparison with the non-sequential control condition which involved passively reading or viewing the stimulus material. In both tasks, this resulted in similar highly significant cerebellar activations in all conditions. For ease of presentation, we report only the conjunction of all 4 contrasts of the experimental conditions in comparison with the control condition. In all comparisons, we used a whole-brain FWE corrected peak threshold *p* < 0.05, voxel size > 10, unless noted otherwise. In both tasks and across all experimental conditions, cerebellar lobules VIII and VI were activated, and also Crus 1 (and Crus 2 in the picture task; Tables 2 and 3; Figs 2 and 3). Additional activations were found during both tasks in the (sub)cortex of the cerebrum, including the precuneus, lateral temporal cortex, the precentral cortex, insula, and lateral frontal cortex (Tables 2 and 3, bottom). In order to test whether the posterior cerebellum is more involved in social sequencing than in routine social and non-social sequencing, we compared the false and true belief conditions with the other experimental (social script and mechanical) conditions. In addition, to test whether false belief understanding is crucial in cerebellar involvement, we also compared the belief conditions among each other. In the story task (Table 2; Fig. 2), this revealed strong activation of the false and true belief conditions in comparison with the social script and mechanical conditions in the bilateral posterior cerebellum (most often Crus 1 and 2), except for true beliefs which did not differ from mechanical events. The two belief conditions did not differ from each other. In addition, the other experimental (i.e., social script and mechanical) conditions as well as all remaining reverse contrasts did not differ.

**Table 2:** Story sequencing: Contrasts from the whole brain analysis.

	x	y	z	voxels	max t
**False Belief > Mechanical**					
R Cerebellum Crus 2	28	−88	−34	81	7.05***
R Cerebellum Crus 1	24	−78	−34		5.15*
L Cerebellum Crus 2	−22	−88	−36	69	5.72**
L Cerebellum Crus 2	−34	−84	−34		5.31*
**False Belief > Social Script**					
R Cerebellum Crus 2	28	−88	−34	154	6.98***
L Cerebellum Crus 1	−22	−84	−32	250	6.78***
L Cerebellum Crus 1	−22	−74	−34		5.51**
L Middle Temporal Gyrus	−54	−42	−4	13	5.42*
L Middle Temporal Gyrus	−52	−16	−12	22	5.78**
L Superior Medial Gyrus	−4	30	46	11	5.42*
**False Belief > True Belief**					
—					
**True Belief > Mechanical**					
—					
**True Belief > Social Script**					
R Cerebellum Crus 2	24	−88	−32	65	5.60**
R Cerebellum Crus 2	30	−84	−38		5.55**
L Cerebellum Crus 2	−24	−84	−34	162	6.50***
L Cerebellum Crus 1	−20	−76	−32		5.37*
R Cerebellum Crus 1	8	−78	−24	18	5.40*
sL Middle Temporal Gyrus	−56	−42	−4	154	5.85**
R Inferior Frontal Opercularis	44	12	34	33	5.53**
L Posterior-Medial Frontal	0	20	48	103	5.74**
L Insula Lobe	−30	22	0	39	6.31***
L Inferior Frontal Orbitalis	−48	44	−6	65	6.72***
**True Belief > False Belief**					
—					
**Conjunction all four Experimental > Control conditions**					
L Middle Occipital Gyrus	−44	−72	10	794	11.90***
L Precuneus	−10	−72	38	828	10.36***
R Precuneus	10	−70	38	673	8.94****
R Middle Temporal Gyrus	44	−70	10	828	8.68***
R Cerebellum VIII	16	−62	−48	217	8.97***
R Cerebellum VIII	24	−56	−50		8.80***
L Cerebellum VI	−32	−54	−30	44	6.86***
R Fusiform Gyrus	22	−54	−12	1635	10.97***
R Cerebellum Crus 1	42	−52	−34		6.82***
L Hippocampus	−22	−40	0	525	7.57***
R Superior Temporal Gyrus	60	−40	22	2549	9.48***
Thalamus: Parietal	−18	−24	16	32	6.26***
L Precentral Gyrus	−2	−18	30	13342	10.58***
L Precentral Gyrus	−58	6	30	26	6.23***
R Insula	40	−12	−8	234	6.29***
R Insula Lobe	44	6	2	1390	9.40***
R Superior Medial Gyrus	10	28	60	30	6.27***
L Superior Frontal Gyrus	−24	42	40	947	7.95***
R Middle Frontal Gyrus	30	46	36	806	7.44***

Notes: Coordinates refer to the MNI (Montreal Neurological Institute) stereotaxic space. Whole-brain analysis thresholded at FWE-corrected *p* < 0.05, with voxel extent ≥ 10. Only the highest peaks of each cluster are shown, except for the cerebellum showing all peaks. L = left, R = right.

*p < 0.05, **p < 0.01, ***p < 0.001 (peak FWE corrected).

**Table 3:** Picture Sequencing: Contrasts from the whole brain analysis

	x	y	z	Voxels	max t
**False Belief > Mechanical**					
R Cerebellum Crus 2	24	−84	−36	175	3.91
R Cerebellum Crus 1	30	−76	−32		3.77
R Cerebellum Crus 1	22	−74	−28		3.73
L Inferior Occipital Gyrus	−34	−78	−10	135	3.80
L Middle Temporal Gyrus	−48	−36	0	254	4.77
L Precentral Gyrus	−34	−6	48	155	4.24
L Posterior-Medial Frontal	−4	12	70	442	4.47
L Middle Frontal Gyrus	−50	20	42	594	4.57
L Inferior Frontal Orbitalis	−46	32	−4	559	5.61**
**False Belief > Social Script**					
L Fusiform Gyrus	−42	−52	−18	144	3.82
vL Middle Frontal Gyrus	−24	−6	50	139	3.97
R Posterior-Medial Frontal	2	8	54	865	5.28**
**False Belief > True Belief**					
—					
**True Belief > Mechanical**					
R Middle Occipital Gyrus	40	−86	16	448	4.05
L Cerebellum Crus 2	−20	−82	−38	2184	5.92***
R Cerebellum Crus 1	28	−72	−36	1105	5.55**
R Cerebellum Crus 2	20	−82	−40		5.07*
R Cerebellum Crus 2	30	−80	−40		4.94*
L Middle Temporal Gyrus	−36	−56	22	690	4.42
L Middle Temporal Gyrus	−54	−38	−2	1147	5.44**
R Middle Temporal Gyrus	50	−32	−10	1161	4.86*
L SupraMarginal Gyrus	−62	−30	32	167	4.13
R Middle Frontal Gyrus	38	6	62	186	5.27**
R Caudate Nucleus	14	6	16	144	4.19
R Inferior Frontal Opercularis	44	8	22	203	4.06
R Inferior Frontal Triangularis	28	24	−4	608	4.94*
vL Superior Medial Gyrus	−4	30	54	864	5.16*
L Inferior Frontal Orbitalis	−44	34	−4	2868	7.67***
**True Belief > Social Script**					
L Cerebellum Crus 2	−24	−86	−36	347	4.49
L Cerebellum Crus 2	−32	−84	−34		4.05
L Cerebellum Crus 2	−16	−80	−48		3.69
L Inferior Temporal Gyrus	−46	−66	−10	339	4.64
L Angular Gyrus	−56	−60	26	179	3.87
L Inferior Parietal Lobule	−32	−48	54	302	4.13
R Middle Temporal Gyrus	62	−38	2	1014	5.15*
L Superior Temporal Gyrus	−66	−34	10	531	4.33
R Insula Lobe	32	22	−2	1169	5.04*
L Middle Frontal Gyrus	−42	24	36	695	4.43
L Insula Lobe	−32	26	2	463	4.95*
R Anterior Cingulate	10	30	24	1977	5.19*
L Superior Frontal Gyrus	−28	62	20	280	4.95
R Superior Frontal Gyrus	28	62	8	209	4.23
**True Belief > False Belief**					
L Cerebellum Crus 2	−22	−80	−38	172	4.04
L Cerebellum Crus 2	−30	−72	−38		4.01
L Cerebellum Crus 2	−14	−74	−38		3.44
**Conjunction of all four Experimental > Control conditions**					
L Cerebellum Crus 2	−40	−78	−40	11	5.47**
R Cerebellum Crus 2	42	−76	−40	35	6.07***
R Cerebellum Crus 1	46	−68	−38		5.87***
L Inferior Parietal Lobule	−44	−70	48	631	10.09***
L Precuneus	−8	−66	32	10553	15.11***
R Angular Gyrus	54	−64	42	332	9.39***
R Cerebellum VIII	16	−64	−48	107	6.75***
R Cerebellum VIII	22	−58	−48		6.62***
R Cerebellum VI	24	−52	−24	162	7.71***
Cerebellar Vermis 4/5	6	−60	−14		5.27**
R Postcentral Gyrus	22	−40	68	290	8.33***
L Precentral Gyrus	−26	−28	58	1581	8.18***
L Insula Lobe	−36	4	12	2952	10.68***
R Insula Lobe	46	6	0	3228	10.06***
L Superior Frontal Gyrus	−22	44	36	1010	8.71***
R Middle Frontal Gyrus	38	48	10	17	5.50**

Notes: Coordinates refer to the MNI (Montreal Neurological Institute) stereotaxic space. Whole-brain analysis thresholded at *p* < 0.001, uncorrected with voxel extent ≥ 10, and reported clusters are clusterwise FWE-corrected *p* < 0.05. The conjunction is thresholded at FWE-corrected *p* < 0.05. Only the highest peaks of each cluster are shown, except for the cerebellum showing all peaks. L = left, R = right.

*p < 0.05, **p < 0.01, ***p < 0.001 (peak FWE corrected).

In the picture task, the results did not show much brain activation at the original FWE corrected threshold. Therefore, we lowered the threshold to an uncorrected voxel-wise threshold of *p* < 0.001, followed by a cluster-wise FWE corrected threshold *p* < 0.05, size > 10 voxels (Table 3) which is less strict, but still conventionally used in fMRI analyses. This exploratory analysis revealed again significant activation of the false and true belief conditions in comparison with the social script and mechanical conditions in the bilateral posterior cerebellum (most often Crus 1 and 2; Table 3; Fig. 3), except for false beliefs which did not differ from social scripts. Although false beliefs did not show stronger activation than true beliefs, the reverse contrast was significant. In addition, the other experimental (i.e., social script and mechanical) conditions as well as all remaining reverse contrasts did not show any differences in the cerebellum, and showed occasional differences only in the occipital and cingulate cortex.

Two additional parametric regression analyses involving participants’ accuracy or AQ scores as covariates did not reveal any significant activation in the cerebellum. Note that this lack of relationship with AQ scores might be due to the low AQ scores overall for our participants.

## Discussion

This study investigated whether the posterior cerebellum is involved in action sequencing and whether social mentalizing plays a crucial role in this. In particular, we tested the hypothesis that the cerebellum is involved in constructing social action sequences that are indicative for or dependent on a person’s mental state. To test this hypothesis, we investigated not only the sequential reconstruction of mechanical or social actions, as was done in earlier research (e.g.^23,24^), but also of social actions which required understanding the mental state of the protagonist (e.g., involving false or true beliefs).

The results strongly supported the hypothesis that the cerebellum is involved in the reconstruction of the order of action sequences^18^. First, comparisons with the passive non-sequential control condition clearly confirmed that the cerebellum is associated with constructing action sequences in general, regardless of whether these sequences are social or non-social, or whether they are routinely or non-routinely executed. Second, the results further supported our hypothesis that specific regions of the posterior cerebellum (mostly Crus 1 and 2) are preferentially activated for constructing novel social sequences of actions involving mental state attributions in comparison with routine action sequences with or without a social element. Specifically, all predicted comparisons between false belief and mechanical events were significant. The differences involving true beliefs and social scripts, which were not part of our main predictions and thus rather exploratory, showed less systematic and strong results. In particular, in each task, there was one comparison involving these conditions that did not reach conventional significance levels (i.e. true belief vs. mechanical stories for the verbal task, and false belief vs. social script stories for the pictorial task). Given their exploratory and non-systematic nature, we do not speculate about the reasons for these weaker results.

In general, our results are in line with earlier research on the cerebellum which showed that reconstructing the correct chronological order of actions reveals strong differences between cerebellar patients and healthy controls^25^, while research lacking an active sequential component did not reveal robust differences^19,31^. Clearly, an active sequencing component is needed to elicit robust cerebellar activation in social mentalizing. This seems to confirm our hypothesis that the cerebellum is generally involved in the prediction and error correction of sequences (by internal models) in the context of movements and cognition, while the activation in specific regions of the posterior cerebellum in the current study, reflects this cerebellar broader function applied on a somewhat different, social cognitive domain. Perhaps there are other subtler differences between cognitive and social-cognitive cerebellar processes which are related to the process of mentalizing, but that is a question for future research.

In addition to cerebellar areas, across both tasks, we also found cortical activation in the precuneus, lateral temporal cortex, the precentral cortex, insula, and lateral frontal areas. The precuneus may be associated with the construction and integration of background scenes of the stories^32^. The activation in the precentral cortex and the dorsolateral prefrontal cortex could be related, as both areas are involved in cognitive manipulation, in this case, sequence generation and attentional selection^33,34^. More research on the effective connectivity between the cerebellum and the cerebral cortex (cf.^35^) might elucidate the precise division of labor in the context of social mentalizing.

We also found that reconstructing the sequence of false beliefs did not activate the cerebellum significantly stronger than true beliefs. Although we found stronger activation in the picture sequencing tasks, this observation was not confirmed in the verbal story task or in a combined analysis across both tasks. This provides an answer to a question left from earlier work (e.g.^25^), namely whether false belief stories trigger cerebellar activity because these stories involve false beliefs or because they involve relatively novel beliefs (regardless of whether true or false beliefs are involved). The present results indicate that true and false belief sequences are treated alike. One explanation is that both stories require mental state inferences, although these are in principle required more for understanding false belief stories than for true belief stories (because false beliefs differ from reality, while true beliefs do not). However, in the present stimulus material, the stories were quite complex, involving highly interacting agents, so that both true and false stories might contain an equal amount of mental state elements to understand the story completely and in an accurate order. Another explanation might be that both false and true belief stories are novel and unfamiliar to the participants, while social scripts reflect well-known action sequences (e.g., brushing your teeth, shopping at the groceries). Still another interpretation is that action sequences with social belief elements versus without beliefs differ in terms of increased complexity in sequencing. However, increased complexity and abstraction is an inherent part of social mentalizing, and thus theoretically it is not immediately evident how to keep these aspects apart. Future theorizing and research might provide an answer to this intriguing question.

This study has an important limitation. There were only 4 trials per condition for the picture sequencing task, leading to somewhat less significant results in the picture task than in the verbal story task. To compensate for this low number of trials and to increase statistical power, we invited more participants for this task. Another way to compensate this was by developing a new analogous verbal sequencing task, for which we created 9 trials in each condition (which is a typical length in clinical research). However, a better solution for future neuroimaging research might be to develop even more material, to obtain increased power to unveil perhaps delicate differences between true and false beliefs in the cerebellum. If no differences would be found again under these conditions, this would strengthen our hypothesis that the social function of the posterior cerebellum differs substantially from the cerebral cortex, where false beliefs typically trigger more activation than true beliefs (e.g., in the TPJ).

Regarding the analyses of our behavioral in-scanner data, we should remark that there were occasional differences between conditions which were not consistent across both tasks, and which did not show any variation during purely behavioral piloting. These differences are presumably due to slower timing of the task under the scanner (due to jittering) and are difficult to interpret. The demonstration of cerebellar involvement in our newly developed social sequencing tasks is an important step towards new clinical implications. The cerebellum has been found to be crucially implicated in the pathophysiological mechanisms subserving a broad range of neuropsychiatric and neurodevelopmental disorders such as ASD, ADHD, depression, and schizophrenia^4–7^. Identification of the role of the cerebellum in social cognition may open very promising avenues for future clinical diagnostics and treatment. Diagnosis of cerebellar dysfunctions in these patients have been neglected for a long time but can hopefully profit in the future from the current extension of the picture sequencing task^26^, as well as from the novel story sequencing task that we developed here. Up till now, such social tests did not constitute an inherent part of the diagnosis and therapy of neuropathological dysfunctions, and patients may have received inadequate support and treatment because of this neglect.

## Supplementary information

Story Sequencing test
