# Supplementary material for: The posterior Cerebellum is involved in constructing Social Action Sequences: An fMRI Study
Source: Sci Rep. 2019 Jul 31;9:11110. doi: 10.1038/s41598-019-46962-7 (PMC6668391; doi:10.1038/s41598-019-46962-7)
Supplement: Supplementary file 1 — Story Sequencing test [file 41598_2019_46962_MOESM1_ESM.docx]

**The posterior Cerebellum is involved in constructing Social Action Sequences:
An fMRI Study**

Elien Heleven, Kim van Dun, and Frank Van Overwalle

Vrije Universiteit Brussel, Belgium

This research was supported by a Grant from the Strategic Research Program from the Vrije Universiteit Brussel (SRP15) awarded to Frank Van Overwalle. Address for correspondence: Elien Heleven, Department of Psychology, Vrije Universiteit Brussel, Pleinlaan 2, B - 1050 Brussel, Belgium; or by e-mail: Elien.Heleven@vub.be.

**Supplementary Material: Story Sequencing test**

The entire test in Dutch and English; all experimental trials (without controls) in French and Italian.

**Story Sequencing test (English)**

Underlined sentence parts indicate the critical elements that signal a false or true belief. The Eprime 2.0 standard program with response box or touch-sensitive / mouse responses is available from Frank Van Overwalle.

Instruction: Please put the sentences of each story in the correct chronological order.

PRACTICE STORIES

Practice 1.

1. While Sylvie takes the key out of her coat pocket, she does not notice that a piece of paper falls out.
2. When the piece of paper almost touches the ground, it is taken away by a gust of wind.
3. The paper flies a few meters high before it slowly comes down.
4. The piece of paper comes to a standstill in the gutter.

Practice 2.

1. Robin puts his dog on a leash and walks out of his house.
2. Robin walks with the dog on the footpath towards the park.
3. Robin lets the leash stretch long in the park.
4. Robin calls his dog, keeps the leash shorter and walks towards the house.

FALSE BELIEF STORIES (adapted from Baron-Cohen et al., 1999)

False belief 1.

1. All of the class took part in the story competition. Emma really wanted to win.
2. Whilst Emma was away from school, the results of the story competition were announced: Alice was the winner.
3. When Emma was back at school, Alice saw Emma and said, "I'm sorry about your story."
4. "What do you mean?" said Emma. "Oh nothing," said Alice.

False Belief 2.

1. Robert had just started at a new school and made friends with some of his class mates, Claire and Andrew.
2. During the break, Claire complained privately to Andrew, "All schools and teachers are horrible."
3. Robert passed by and asked Claire, "My Mom is a teacher at this school. Do you want to come to my home and play Monopoly?"
4. Claire said, "Sorry, I’m not feeling very well."

False Belief 3.

1. Mike was in one of the cubicles in the toilets at school. Joe and Peter were at the sinks nearby and did not notice Mike.
2. Joe said, "You know that new boy in the class, his name is Mike. Doesn't he look really weird!"
3. Mike came out of the cubicles.
4. Peter said, "Oh hello Mike, are you going to play football now?"

False Belief 4.

1. Kim helped her Mum make the special pie with apple hidden in the dough for her uncle Tom when he came to visit.
2. When uncle Tom arrived, Kim carried the pie out of the kitchen.
3. Kim showed the pie to uncle Tom and said proudly, "I made the pie just for you.”
4. "Mmm," said Uncle Tom, "that pie looks lovely. I love pies, except for apple, of course!"

False Belief 5.

1. James bought Richard’s brother a toy airplane for his birthday, while Richard was on a holiday.
2. A few months later, James and Richard were playing with the toy airplane.
3. James accidentally dropped the toy airplane.
4. "Don't worry," said Richard to James, "I never liked the toy airplane anyway. Someone gave it to my brother for his birthday."

False Belief 6.

1. Sally, who has short blonde hair, was at her Aunt Carol's house when the doorbell rang.
2. At the door was Mary, a neighbor.
3. Mary said, "Hello," and took off her glasses. Then she looked at Sally and said, "Oh, I don't think I've met this little boy. What's your name?"
4. Aunt Carol said, "This little girl is Sally."

False Belief 7.

1. The teacher said at the start of her class, "One of the boys in our class, Simon, is very seriously ill."
2. The class were all very sad and quiet when a little girl, Becky, arrived late.
3. "Have you heard my new joke about sick people?" Becky asked.
4. The teacher did not laugh and said to Becky, "Sit down and get on with your work."

False Belief 8.

1. Tim was having breakfast in a restaurant.
2. Tim spilt his coffee on the floor by accident, and looked for someone to clean up the mess.
3. Tim went up to Jack the waiter, who was in the kitchen.
4. Jack asked to Tim, "Do you want to pay for the coffee?”

False Belief 9.

(Not used)

1. ~~Jill had just moved into her new house without curtains.~~
2. ~~Jill went shopping, bought some new curtains, and put them up.~~
3. ~~Her best friend Lisa came round to visit the house and said, "Oh, those old curtains do not fit the room! You should buy new ones!"~~
4. ~~Jill asked, "Do you like the rest of my house?"~~

False Belief 10.

1. Helen's mum was having a surprise party for Helen's birthday. She invited Nicky and said, "Don't tell anyone, especially Helen!"
2. The day before the party Nicky and Helen were playing together and Nicky ripped her new dress.
3. "Oh!" said Nicky, "I was going to wear this to your party."
4. "What party?" asked Helen. "Come on," said Nicky, "let's go and see if my mum can mend the tear."

TRUE BELIEF STORIES (adapted from Baron-Cohen et al., 1999)

True Belief 1.

1. All of the class took part in the poetry competition. Jane really wanted to win.
2. While Jane was at school, the results of the poetry competition were announced: Mary was the winner.
3. After school, Jane bumped into Mary. Mary said, "I'm sorry about your story. How are you feeling?"
4. "Horrible!" said Jane. "I understand," said Mary.

True Belief 2.

1. David had just started at a new school and made friends with some of his class mates, Jeff and Mike.
2. During the break, Jeff complained to Mike and David, "All schools and teachers are horrible."
3. David stood nearby and asked Jeff, "My Mom works in a candy store, do you want some?"
4. Jeff said, "Sorry, I’m not feeling very well."

True Belief 3.

1. John was in one of the cubicles in the toilets at school. Sam and Eddy were at the sinks nearby.
2. Sam said, "Hey new boy, John, are you in there?"
3. John came out of the cubicles.
4. Peter said, "Oh, hi John. Are you going to play football now?"

True Belief 4.

1. Kate helped her Mum make the special pie with fruit on top of the dough for her neighbor when he came to visit.
2. When the neighbor arrived, Kate carried the pie out of the kitchen.
3. Kate showed the pie to the neighbor and said proudly, "I made the pie just for you."
4. "Mmm," said her neighbor, "that pie looks lovely — I love pies, especially fruit ones!"

True Belief 5.

1. Simon bought Robert’s brother a toy car for his birthday, while Robert was also at the birthday party.
2. A few months later, Simon and Robert were playing with the toy car.
3. Simon dropped the toy car.
4. "Don't worry," said Robert, "it was only an accident."

True Belief 6.

1. Jill, who has short brown hair, was at her Uncle Ted's house when the doorbell rang.
2. At the door was Mrs. Smith, a neighbor.
3. Mrs. Smith said, "Hello," and put on her glasses. Then she looked at Jill and said, "Oh, I don't think I've met this little girl. What's your name?"
4. Uncle Ted said, "This little girl is Jill".

True Belief 7.

1. The teacher told her class at the start of the class, "You all know George in our class, well, he is very seriously ill."
2. The class were all very sad and quiet. A little girl, Amy, started to cry.
3. "Sorry for crying, I've just been to the dentist," Amy said.
4. The teacher said to Amy, "Sit down and get on with your work."

True Belief 8:

1. Alan was having a drink in a restaurant.
2. Alan spilt his coke on the floor by accident, and looked for someone to clean up the mess.
3. Alan went up to Edward the waiter, who was standing in the restaurant.
4. Edward asked to Alan, "Would you like me to mop the spilled coffee up?"

True Belief 9.

(Not used)

1. ~~Michelle had just moved into her new house without a rug for the bedroom.~~
2. ~~Michelle went shopping with her friend Samantha, bought a new rug for her bedroom, and put it down~~
3. ~~Her best friend, Samantha, came round to visit the house and said, "Oh, your new rug does not fit the room! You should buy another one!"~~
4. ~~Michelle asked, "Do you like the rest of the house?"~~

True Belief 10.

1. Annette's mum was having a party for Annette's birthday. She invited Bridget and said to her, "Please tell all Annette’s friends!"
2. The day before the party Annette and Bridget were playing together and Bridget ripped her new shirt.

"Oh!" said Bridget, "I was going to wear this to your party."

1. "Maybe you still can," said Annette. "Come on,” said Bridget, “let's go and see if my mum can mend the tear.”

MECHANICAL (Novel, best translation from Dutch)

Mechanical 1

1. Pieter rode his bike quickly round the bend.
2. The bicycle tire hit a pinecone that rolled away due to the impact.
3. The pinecone began to roll faster and faster down the slope in the direction of the stream.
4. The pinecone drifted away on the fast and wildly flowing water of the stream.

Mechanical 2

1. The speedboat in which Erik was racing flew against a rock at high speed.
2. The speedboat hit the rock with a loud cracking sound, and a piece of rock broke off and flew away.
3. The piece of rock hurtled against another boat.
4. The flying piece of rock hit the hull of the other boat, and made a hole in it.

Mechanical 3

1. A strong wind rushed through the window into Michael's room.
2. The door slammed with a loud bang due to the gust of wind.
3. The shock caused a glass vase to vibrate on the table right next to the door.
4. The vase fell off the table and onto the floor, and smashed into a thousand pieces.

Mechanical 4

1. The door of the cabinet closed with great force because of the wind.
2. On the cabinet was a black ball that began to roll due to the shock.
3. The black ball rolled off the cabinet and onto the floor in the direction of the table.
4. The black ball rolled under the table and came to a standstill against the farthest table leg.

Mechanical 5

1. The sun rose above the horizon.
2. The sun was high in the sky.
3. The sun began to sink behind the dunes.
4. The sun sank completely behind the horizon.

Mechanical 6

(Not used)

Mechanical 7

1. There was no cloud in the sky.
2. In the afternoon the clouds began to gradually increase.
3. Later it started to rain.
4. After days of rain, the brook overflowed.

Mechanical 8

1. The volcano was inactive for years.
2. The volcano started to emit lava.
3. The hot lava flowed down the mountainside.
4. The hot lava reached the sea.

Mechanical 9

1. All the cars went up the road early in the morning.
2. The traffic on the highway flowed very smoothly, but 10 minutes later it began to slow down.
3. The cars started to crawl at a walking pace, until they came to a standstill in a traffic jam.
4. The traffic jam slowly dispersed and the traffic finally got moving again.

Mechanical 10

1. There was suddenly a small crack on the edge of the Antarctic ice shelf.
2. After a few days the crack increased.
3. An iceberg tore off from the ice shelf.
4. The iceberg drifted off into the sea.

SOCIAL SCRIPTS (Novel, best translation from Dutch)

Social Script 1

(Not used)

1. ~~Tom walked into the supermarket and took a shopping basket at the entrance.~~
2. ~~Tom took pasta, tomato sauce and cheese from the racks and put them in the basket.~~
3. ~~At the cash register, Tom put all the things in his bag and he paid with his bank card.~~
4. ~~When leaving the store, Tom left the shopping basket.~~

Social Script 2

1. Silke went to the fitting rooms and waited for her turn.
2. When a fitting room was available, Silke told the shop assistant how many pieces of clothing she wanted to try.
3. Silke tried two shirts and a skirt. She decided to buy the skirt.
4. After leaving the fitting room, Silke left the two shirts with the shop assistant.

Social Script 3

1. Walter took his mobile out of his pocket and started the device.
2. Walter keyed in the number of a friend he wanted to ask something.
3. When Walter’s friend did not answer, he left a voicemail in which he asked him to call back.
4. Walter hung up and put his mobile back in his pocket.

Social Script 4

1. Yasmine bought a birthday card for her friend at the newsagent.
2. Yasmine wrote a sweet message on the card and put it in an envelope.
3. Yasmine closed the envelope, wrote the address and stuck a stamp on it.
4. Yasmine went to the mailbox and put the card in the slot.

Social Script 5

1. Katja was looking for some pieces of furniture for her apartment on a website.
2. When Katja found what she was looking for, she selected the furniture and clicked to pay.
3. Katja chose a payment method and filled in the requested data.
4. The website indicated that Katja's purchase was successful.

Social Script 6

1. Kristian walked into the library and searched on the shelves for the book he wanted to read.
2. When Kristian found the book, he took it to the check-out desk.
3. At the check-out desk, Kristian scanned his library card and the book.
4. Kristian took the book home.

Social Script 7

1. Kristof put a pot of water on the stove.
2. When the water started to boil, Kristof put pasta in the pot.
3. Kristof waited a few minutes until the pasta was done.
4. Kristof drained the water and ate the pasta.

Social Script 8

1. Ben went into the movie theater and watched the movie of the day.
2. Ben bought a ticket for the movie he wanted to see.
3. Before Ben entered the auditorium, he bought chips and a drink.
4. Ben sat down on a chair and watched the movie.

Social Script 9

1. Steven looked at the bus schedule. When it was nearly time for the bus to arrive, he walked to the bus stop.
2. When the bus arrived, Steven got in and scanned his bus card.
3. When the bus was almost at the right stop, Steven pressed the bell.
4. Steven got out of the bus and walked to his destination.

Social Script 10

1. Joris sat at a table in a restaurant and looked at the menu.
2. Joris summoned the waiter and ordered a dish and a drink.
3. When Joris had finished eating, he summoned the waiter and asked for the check.
4. Joris paid for the food and drink and gave a small tip.

CONTROLS (Novel baseline for fMRI, translated from Dutch)

Instruction: Read the sentences and answer the question

Control 1

1. Sandra saw the moon in a clear sky
2. Gradually clouds came over, darkening the night
3. The clouds occasionally drifted in front of the moon
4. The clouds covered the sky completely, the moon was no longer visible

Was the moon visible all night?

Correct answer: No

Control 2

1. It’s pouring with rain
2. Several drops splash against the window
3. Slowly the drops slide down the window
4. The drops end up in a puddle on the windowsill

Are the drops sliding down the window?

Correct answer: Yes

Control 3

1. Daan accidentally bumps a glass of water with his hand
2. As a result, the glass falls over
3. The glass breaks when it hits the table
4. The water spreads over the table

Was there water in the glass?

Correct answer: Yes

Control 4

1. A strong wind blows through the trees
2. The leaves on the trees begin to move
3. A small branch creaks, tears and breaks off
4. The branch falls from the tree to the ground

Is the branch still hanging on the tree?

Correct answer: No

Control 5

1. Roan throws the frisbee towards the park
2. The frisbee flies through the air over a few benches
3. The frisbee get caught between the branches of the trees
4. The frisbee drops a little but stays hanging in the branches

Does Roan throw the frisbee on the floor?

Correct answer: No

Control 6

1. The warm, humid air rises
2. The humid airflow forms threatening clouds
3. The clouds become heavier and there is a thunderstorm
4. The lightning illuminates the sky

Are threatening clouds forming?

Correct answer: Yes

Control 7

1. The pan is standing on a fire that is getting hotter and hotter
2. The water starts to boil so that the potatoes cook
3. Almost all water has evaporated
4. The potatoes burn at the bottom of the pan

Will the potatoes taste good?

Correct answer: No

Control 8

1. Chloe puts the frozen pizza in the oven
2. The pizza defrosts slowly because of the heat
3. The pizza starts to warm up and the cheese starts to melt
4. The cheese has melted and the pizza has a crispy crust

Is the pizza cooked?

Correct answer: Yes

Control 9

1. The soap sprays out of the container and ends up in the water
2. The soap slowly spreads in the water
3. Rita turns on the tap to allow extra hot water to run
4. The soap starts to foam

Does the running water make the soap foam?

Correct answer: Yes

**Story Sequencing test (Nederlands)**

Instructies: Zet de zinnen van de verhalen in de juiste chronologische volgorde.

PRACTICE STORIES

Practice 1

1. Terwijl Sylvie de sleutel uit haar jaszak neemt, merkt ze niet dat er een stukje papier uit valt.
2. Wanneer het papiertje bijna de grond raakt, wordt het meegenomen door een windvlaag.
3. Het papiertje vliegt enkele meters hoog voordat het langzaam naar beneden komt.
4. Het papiertje komt tot stilstand in de goot.

Practice 2

1. Robin doet zijn hond een leiband om en wandelt zijn huis uit.
2. Robin wandelt met de hond over het voetpad richting het park.
3. Robin laat de leiband lang uitrekken in het park.
4. Robin roept zijn hond korter bij zich en wandelt richting huis.

FALSE BELIEF STORIES (adapted from Baron-Cohen et al., 1999)

False Belief 1

1. De hele klas nam deel aan de verhalencompetitie. Emma wilde heel graag winnen.
2. Terwijl Emma afwezig was van school, werden de resultaten van de verhalencompetitie aangekondigd: Alice was de winnaar.
3. Toen Emma weer op school was, zag Alice Emma en zei: "Het spijt me van je verhaal."
4. "Wat bedoel je?" zei Emma. "Oh niets," zei Alice.

False Belief 2

1. Robert zat net op een nieuwe school en werd bevriend met enkele van zijn klasgenoten, Claire en Andres.
2. Tijdens de pauze klaagde Claire uitsluitend tegen Andres, "Alle scholen en leraren zijn vreselijk. "
3. Robert kwam even later langs en vroeg Claire, "Mijn moeder is een lerares op deze school. Wil je met ons mee naar huis komen en Monopoly spelen?"
4. Claire zei "Sorry, Ik voel me niet goed."

False Belief 3

1. Mike zat in een van de toilethokjes op school. Jan en Peter waren in de buurt van de wasbakken en merkten Mike niet op.
2. Mike hoorde Jan tegen de anderen zeggen: "Je kent die nieuwe jongen in de klas, hij heet Mike. Ziet hij er niet raar uit?"
3. Mike kwam uit het toilethokje.
4. Peter zei: "Oh hallo Mike, ga je mee voetballen?"

False Belief 4

1. Voor het bezoek van haar oom Tom, hielp Kim haar moeder een speciale taart maken met appel verstopt in het deeg.
2. Toen oom Tom arriveerde, haalde Kim de taart uit de keuken.
3. Kim liet de taart zien aan oom Tom en zei trots "Ik maakte deze taart voor jou.”
4. "Mmm", zei oom Tom, "Die taart ziet er goed uit. Ik hou van taarten, met uitzondering van appeltaart, natuurlijk!"

False Belief 5

1. Jacob gaf de broer van Rik een speelgoedvliegtuigje voor zijn verjaardag, terwijl Rik op vakantie was.
2. Een paar maanden later, waren Jacob en Rik aan het spelen met het speelgoedvliegtuigje.
3. Toen Jacob en Rik samen speelden, liet Jacob per ongeluk het speelgoedvliegtuigje vallen.
4. "Maak je geen zorgen", zei Rik tegen Jacob, "Ik heb het speelgoedvliegtuigje toch nooit leuk gevonden. Iemand gaf hem aan mijn broer voor zijn verjaardag."

False Belief 6

1. Sarah, die kort blond haar heeft, was in het huis van haar tante Karen toen werd aangebeld.
2. Het was Marie, een buurvrouw.
3. Marie zei "Hallo" en nam haar bril af. Ze keek naar Sarah en zei: "Oh, ik denk niet dat ik deze kleine jongen al heb ontmoet. Wat is je naam?"
4. Tante Karen zei: “Dit kleine meisje is Sarah."

False Belief 7

1. De leerkracht vertelde aan het begin van haar les "Een van de jongens in onze klas, Simon, is ernstig ziek".
2. De leerlingen waren allemaal erg verdrietig en stil, toen een klein meisje, Becky, te laat binnen kwam.
3. "Heb je mijn nieuwe grap over zieke mensen al gehoord?" vroeg Becky.
4. De lerares lachte niet en zei aan Becky "Ga zitten en begin met je werk."

False Belief 8

1. Tim was aan het ontbijten in een restaurant.
2. Tim morste per ongeluk zijn koffie op de vloer en zocht iemand om de rotzooi op te kuisen.
3. Tim ging naar Joris, de ober, die in de keuken was.
4. Joris vroeg aan Tim, "Wenst u te betalen voor de koffie?”

False Belief 9

(Not used)

1. ~~Julie was net verhuisd naar een nieuw huis zonder gordijnen.~~
2. ~~Julie ging vlak na de verhuis winkelen, kocht een aantal nieuwe gordijnen, en hing ze op.~~
3. ~~Haar beste vriendin Lisa kwam langs om het huis te bezoeken en zei: "Ach, die oude gordijnen passen niet in de kamer! Je moet nieuwe kopen!"~~
4. ~~Julie vroeg: "Wil je de rest van mijn huis zien?"~~

False Belief 10

1. De moeder van Heleen organiseerde een verrassingsfeest voor Heleens verjaardag. Ze nodigde Nicky uit en zei: "Niemand iets vertellen, vooral Heleen niet!"
2. De dag voor het feest speelden Nicky en Heleen samen toen Nicky haar nieuwe jurk scheurde.
3. "Oh!" zei Nicky, "ik wou dit op je feestje dragen."
4. "Welk feest?" vroeg Heleen. "Kom," zei Nicky. "Laten we mijn moeder vragen of ze de scheur kan repareren."

TRUE BELIEF STORIES (adapted from Baron-Cohen et al., 1999)

True Belief 1

1. De hele klas nam deel aan de poëziewedstrijd. Janne wilde heel graag winnen.
2. Terwijl Janne op school was, werden de resultaten van de poëziewedstrijd aangekondigd: Marie was de winnaar.
3. Later op school botste Janne op Marie. Marie zei "Het spijt me van je gedicht. Hoe voel je je?".
4. "Ik voel me verschrikkelijk! "Zei Janne," Ik begrijp het" zei Marie.

True Belief 2

1. David was net begonnen op een nieuwe school en werd bevriend met enkele van zijn klasgenoten, Jeff en Mike.
2. Tijdens de pauze klaagde Jeff bij Mike: "Alle scholen en leraren zijn vreselijk. "
3. David stond in de buurt en vroeg Jeff, "Mijn moeder werkt in een snoepwinkel, wil je wat snoep?"
4. Jeff zei "Sorry, Ik voel me niet goed."

True Belief 3

1. Johan zat in een van de toilethokjes op school. Sam en Eddy waren in de buurt van de wasbakken.
2. Sam zei "Hallo nieuwe jongen, Johan, ben je daarbinnen?"
3. Johan kwam uit het toilethokje.
4. Eddy zei: "Oh, hoi Johan. Ga je mee voetballen?"

True Belief 4

1. Voor het bezoek van haar buurman hielp Kaat haar moeder een speciale taart maken met fruit bovenop het deeg.
2. Toen de buurman arriveerde, haalde Kaat de taart uit de keuken.
3. Kaat liet de taart aan de buurman zien en zei trots "Ik maakte deze taart voor jou."
4. "Mmm", zei haar buurman, "Die taart ziet er goed uit - Ik hou van taarten, vooral fruittaarten!"

True Belief 5

1. Simon gaf Roberts broer een speelgoedauto voor zijn verjaardag, terwijl Robert ook op het verjaardagsfeest was.
2. Een paar maanden later speelden Simon en Robert samen met de speelgoedauto
3. Terwijl Simon en Robert samen speelden, liet Simon de speelgoedauto vallen.
4. "Maak je geen zorgen", zei Robert, "Het was maar een ongeluk".

True Belief 6

1. Julie, die kort bruin haar heeft, was in het huis van haar oom Tony toen er werd aangebeld.
2. Het was mevrouw Smet, een buurvrouw.
3. Mevrouw Smet zei: "Hallo," en deed haar bril aan. Ze keek naar Julie en zei: "Oh, ik denk niet dat ik dit kleine meisje al heb ontmoet. Wat is je naam?"
4. Oom Tony zei "Dit kleine meisje is Julie".

True Belief 7

1. De lerares vertelde haar klas aan het begin van de les, "Jullie kennen allemaal Gert van onze klas, hij is heel erg ziek".
2. De leerlingen waren allemaal erg verdrietig en stil. Een meisje, An, begon te huilen.
3. "Sorry voor het huilen, ik ben net naar de tandarts geweest" zei An.
4. De lerares zei tegen An: "Ga zitten en doe voort met je werk."

True Belief 8

1. Alain bestelde een cola in een restaurant.
2. Alain morste per ongeluk zijn cola op de grond en zocht iemand om de rotzooi op te kuisen.
3. Alain ging naar Edward, de ober, die in het restaurant stond.
4. Edward vroeg aan Alain, "Zal ik de gemorste cola opdweilen?"

True Belief 9

(Not used)

1. ~~Michelle was net verhuisd naar haar nieuw huis zonder een tapijt in de slaapkamer.~~
2. ~~Michelle ging vlak na de verhuis winkelen met haar vriendin Samantha, kocht een nieuw tapijt en legde het neer in de slaapkamer.~~
3. ~~Samantha bekeek de slaapkamer en zei: "Oh, je nieuwe tapijt past niet in de slaapkamer! Je moet een ander kopen!"~~
4. ~~Michelle vroeg: "Vind je de rest van het huis leuk?"~~

True Belief 10

1. De moeder van Annabelle organiseerde een groot feest voor de verjaardag van Annabelle. Ze nodigde Barbara uit en zei haar. "Vertel het alsjeblieft aan alle vrienden van Annabelle!"
2. De dag voor het feest speelden Annabelle en Barbara samen, toen Barbara haar nieuwe kleed scheurde.
3. "Oh!" zei Barbara, "Ik wou dit op je feestje dragen."
4. "Misschien kan dat nog" zei Annabelle. "Kom," zei Barbara "Laten we mijn moeder vragen of ze de scheur kan herstellen.”

MECHANICAL (Novel, original in Dutch)

Mechanical 1

1. Pieter reed met zijn fiets snel door de bocht.
2. De fietsband stootte tegen een dennenappel, die wegrolde door de schok.
3. De dennenappel begon sneller en sneller van de helling te rollen in de richting van de beek.
4. De dennenappel dreef weg op het snelle en wild golvende water van de beek.

Mechanical 2

1. De speedboot waar Erik mee racete, vloog met hoge snelheid uit de bocht tegen een rots.
2. De speedboot raakte de rots met luid gekraak, en er brak een stuk steen af dat wegvloog.
3. Het stuk steen vloog met duizelingwekkende snelheid af op een andere boot.
4. Het vliegende stuk steen stootte tegen de romp van de andere boot, en maakte er een gat in.

Mechanical 3

1. Een felle wind joeg door het raam in de kamer van Michael.
2. Door de windstoot vloog de deur met een luide slag dicht.
3. De schok bracht een glazen vaas aan het trillen op de kast vlak naast de deur.
4. De vaas viel omver van de kast op de grond, waarna ze in duizenden stukjes uiteen spatte.

Mechanical 4

1. Door de wind klapte de deur van de kast met grote kracht dicht.
2. Op de kast lag een zwarte bal die door de schok begon te rollen.
3. De zwarte bal rolde van de kast op de grond in de richting van de tafel.
4. De zwarte bal rolde onder de tafel door en kwam tot stilstand tegen de verste tafelpoot.

Mechanical 5

1. De zon kwam net op aan de horizon.
2. De zon stond hoog aan de hemel.
3. De zon begon te zakken achter de duinen.
4. De zon zakte helemaal achter de horizon.

Mechanical 6

(Not used)

Mechanical 7

1. Er was geen wolkje aan de hemel.
2. In de namiddag begon de bewolking geleidelijk toe te nemen.
3. Later begon het te regenen.
4. Na dagenlange regen, liep de beek over.

Mechanical 8

1. De vulkaan was jarenlang inactief.
2. De vulkaan begon lava uit te stoten.
3. De hete lava vloeide van de bergwand.
4. De hete lava bereikte de zee.

Mechanical 9

1. Alle auto’s reden ’s ochtends vroeg de weg op.
2. Het verkeer op de snelweg ging heel vlot, maar een tiental minuten later begon de snelheid te verminderen.
3. De auto’s begonnen stapvoets te rijden, tot ze tot stilstand kwamen in de file.
4. De file loste langzaam op en het verkeer kwam eindelijk weer in beweging.

Mechanical 10

1. Op de Zuidpool was plots een kleine scheur in de ijsmassa te zien.
2. Na enkele dagen werd de scheur groter.
3. De ijsmassa scheurde zich af van het vasteland.
4. De ijsmassa dreef verder de zee in.

SOCIAL SCRIPTS (Novel, original in Dutch)

Social Script 1

(Not used)

1. ~~Tom liep de supermarkt binnen en nam een winkelmandje bij de ingang.~~
2. ~~Tom nam pasta, tomatensaus en kaas uit de rekken en legde ze in het mandje.~~
3. ~~Bij de kassa stopte Tom alle spullen in zijn tas en hij betaalde met zijn bankkaart.~~
4. ~~Bij het verlaten van de winkel, liet Tom het winkelmandje achter.~~

Social Script 2

1. Silke schoof aan bij de paskamers en wachtte op haar beurt.
2. Toen er een paskamer vrij kwam, vertelde Silke de winkelbediende hoeveel kledingstukken ze wou passen.
3. Silke probeerde twee shirts en een rok. Ze besloot de rok te kopen.
4. Bij het verlaten van de paskamer, liet Silke de twee shirts achter bij de winkelbediende.

Social Script 3

1. Walter nam zijn gsm uit zijn broekzak en activeerde het toestel.
2. Walter toetste het nummer in van een kameraad die hij iets wou vragen.
3. Toen Walters’ kameraad niet opnam, liet hij een voicemail achter waarin hij vroeg hem terug te bellen.
4. Walter hing op en stak zijn gsm terug weg.

Social Script 4

1. Yasmine kocht een verjaardagskaart voor haar vriendin bij de krantenwinkel.
2. Yasmine schreef een lieve boodschap op de kaart en stak ze in een enveloppe
3. Yasmine sloot de enveloppe, schreef het adres en kleefde er een postzegel op.
4. Yasmine liep naar de postbus en stak het kaartje in de gleuf.

Social Script 5

1. Katja zocht enkele meubelstukken op een website om haar appartement in te richten.
2. Toen Katja vond wat ze zocht, selecteerde ze de meubels en klikte op betalen.
3. Katja koos een betaalmethode en vulde de gevraagde gegevens in.
4. De website gaf aan dat Katja’s aankoop succesvol was.

Social Script 6

1. Kristian wandelde de bibliotheek binnen en zocht in de rekken naar het boek dat hij wilde lezen.
2. Toen Kristian het boek vond, nam hij het mee naar de uitleenbalie.
3. Aan de uitleenbalie, scande Kristian zijn bibliotheekpasje en het boek.
4. Kristian nam het boek mee naar huis.

Social Script 7

1. Kristof zette een kookpot met water op het vuur
2. Wanneer het water begon te koken, deed Kristof pasta in de kookpot.
3. Kristof wachtte enkele minuten tot de pasta gaar was.
4. Kristof goot het water af en at de pasta op.

Social Script 8

1. Ben ging de bioscoop binnen en keek naar het filmaanbod van de dag.
2. Ben kocht een ticket voor de film die hij wou zien.
3. Voordat Ben de zaal binnen ging, kocht hij chips en een drankje.
4. Ben ging op een stoeltje zitten en keek naar de film.

Social Script 9

1. Steven bekeek het tijdsschema van de bus. Wanneer de bus bijna aankwam, wandelde hij naar de bushalte.
2. Toen de bus aankwam, stapte Steven op en scande hij zijn buskaart
3. Toen de bus bijna aan zijn halte was, drukte Steven op de bel.
4. Steven stapte uit de bus en wandelde verder naar zijn bestemming.

Social Script 10

1. Joris zat aan een tafeltje in een restaurant en bekeek de menukaart.
2. Joris riep de ober en bestelde een gerecht en een drankje.
3. Toen Joris klaar was met eten, riep hij de ober en vroeg de rekening.
4. Joris betaalde voor het eten en drinken en gaf een kleine fooi.

CONTROLS (Novel baseline for fMRI, original in Dutch)

Instructie: Lees de zinnen en beantwoord de vraag.

Controle 1

1. Sandra zag de maan aan de heldere hemel staan
2. Stilaan kwamen er wolken aangedreven die de nachtlucht donkerder maakten
3. De wolken dreven af en toe voor de maan
4. Het wolkendek trok helemaal dicht, de maan was niet meer te zien

Was de maan de hele nacht zichtbaar?

Correct antwoord: Neen

Controle 2

1. De regen valt met bakken uit de lucht
2. Verschillende druppels spatten tegen het raam
3. Langzaam glijden de druppels langs het raam naar beneden
4. Op de vensterbank eindigen de druppels in een waterplas

Glijden de druppels langs het raam naar beneden?

Correct antwoord: Ja

Controle 3

1. Daan stoot per ongeluk met zijn hand tegen het glas water
2. Door de tik valt het glas om
3. Het glas breekt wanneer het de tafel raakt
4. Het water verspreidt zich over de tafel

Zat er water in het glas?

Correct antwoord: Ja

Controle 4

1. Een felle wind waait door de bomen
2. De bladeren in de bomen beginnen te bewegen
3. Een kleine tak kraakt, scheurt en breekt af
4. De tak valt uit de boom op de grond

Hangt de tak nu nog aan de boom?

Correct antwoord: Neen

Controle 5

1. Roan gooit de frisbee in de richting van het park
2. De frisbee suist door de lucht over enkele banken
3. De frisbee blijft haken tussen de takken van de bomen
4. De frisbee valt nog een beetje naar beneden maar blijft hangen in de takken

Gooit Roan de frisbee op de grond?

Correct antwoord: Neen

Controle 6

1. De warme vochtige lucht stijgt
2. De vochtige luchtstroom vormt dreigende wolken
3. De wolken worden zwaarder en er ontstaat onweer
4. Een bliksemschicht verlicht de hemel

Vormen zich dreigende wolken?

Correct antwoord: Ja

Controle 7

1. De pan staat op een steeds warmer wordend vuur
2. Het water begint te koken zodat de aardappels garen
3. Bijna al het water is verdampt
4. De aardappels verbranden op de bodem van de pot

Zullen de aardappels lekker smaken?

Correct antwoord: Neen

Controle 8

1. Chloe zet de bevroren pizza in de oven
2. Door de hitte ontdooit de pizza langzaam
3. De pizza begint op te warmen en de kaas begint te smelten
4. De kaas is gesmolten en de pizza heeft een knapperige korst

Is de pizza gebakken?

Correct antwoord: Ja

Controle 9

1. De zeep spuit uit de bus en komt in het water terecht
2. De zeep verspreidt zich langzaam in het water
3. Rita zet de kraan open om extra warm water te laten lopen
4. De zeep begint te schuimen

Doet het lopende water de zeep schuimen?

Correct antwoord: Ja

**Story Sequencing test (FR)**

Instruction : Mettre les phrases des histoires dans l'ordre chronologique correct.

PRACTICE STORIES

Practice 1

1. Alors que Sylvie retire la clé de sa poche, elle ne remarque pas qu'un bout de papier s’en échappe.
2. Lorsque le papier arrive presqu’au sol, il est emporté par une rafale de vent.
3. Le papier s’envole à plusieurs mètres de hauteur avant de redescendre lentement.
4. Le papier s'arrête dans le caniveau.

Practice 2

1. Robert met la laisse autour du cou de son chien et sort de chez lui.
2. Robert se promène sur le trottoir avec son chien en direction du parc.
3. Une fois dans le parc, Robert relâche la laisse.
4. Robert rappelle son chien et repart chez lui.

FALSE BELIEF STORIES (adapted from Baron-Cohen et al., 1999; translated from Dutch)

False Belief 1

1. Toute la classe participe au concours de contes. Emma voudrait vraiment gagner.
2. Les résultats du concours sont annoncés alors qu’Emma est absente. La gagnante est Alice.
3. Quand Emma revient à l'école, Alice lui dit : « Je suis désolée pour ton histoire ».
4. « Que veux-tu dire ? » demande Emma. « Oh rien », répond Alice.

False Belief 2

1. Robert s’est inscrit dans une nouvelle école et s'est lié d'amitié avec ses camarades de classe Claire et André.
2. Pendant la pause, Claire se plaint uniquement auprès d’André en disant « Toutes les écoles et tous les professeurs sont épouvantables.»
3. Robert les rejoint un peu plus tard et dit à Claire : « Ma mère est professeur dans cette école. Tu veux rentrer avec nous et venir jouer au Monopoly ? »
4. Claire dit : « Désolée », je ne me sens pas bien".

False Belief 3

1. Michel est aux toilettes de l’école. Pierre et Jean sont près des lavabos et n’ont pas remarqué la présence de Michel.
2. Michel entend Jean dire : « Tu vois ce nouveau garçon en classe, il s'appelle Michel. Il a l'air bizarre, non ? »
3. Michel sort des toilettes.
4. Pierre dit : « Oh bonjour Michel, tu viens jouer au football avec nous ? »

False Belief 4

1. Avant l’arrivée de son oncle Michel, Marie aide sa mère à faire une tarte qui contient des morceaux de pomme cachés.
2. Quand son oncle arrive, Marie va chercher la tarte à la cuisine.
3. Marie montre la tarte à son oncle Michel et lui dit fièrement : « J'ai fait cette tarte spécialement pour toi. »
4. «Mmmm », dit oncle Michel « Cette tarte a l’air bonne. J’adore les tartes, sauf bien sûr la tarte aux pommes »

False Belief 5

1. Alors qu’Antoine est en vacances, Jean offre un avion en plastique au frère d’Antoine pour son anniversaire.
2. Quelques mois plus tard, Jean et Antoine jouent avec l’avion.
3. Alors que Jean et Antoine sont en train de jouer, Jean laisse tomber l’avion accidentellement.
4. «  Ne t’inquiète pas », dit Antoine à Jean « Je n’ai jamais aimé cet avion. Quelqu’un l’a donné à mon frère pour son anniversaire ».

False Belief 6

1. Sarah, qui porte de courts cheveux blonds est en visite chez sa tante Carine quand on sonne à la porte.
2. C'est Marie, une voisine.
3. Marie dit « bonjour » et enlève ses lunettes. Elle regarde Sarah et dit : « Oh, je ne crois pas avoir déjà rencontré ce petit garçon. Comment t’appelles-tu ? »
4. Tante Carine dit : « Cette petite fille s’appelle Sarah ».

False Belief 7

1. Avant de commencer le cours, le professeur dit : « L’un de vos camarades de classe, Simon, est gravement malade ».
2. Les élèves sont tous tristes et silencieux quand Marie arrive en classe, en retard.
3. « Avez-vous déjà entendu ma nouvelle blague sur les malades ? » demande Marie.
4. Le professeur ne rit pas et dit à Marie : « Va t’asseoir et mets-toi au travail.»

False Belief 8

1. Antoine prend le petit déjeuner dans un restaurant.
2. Antoine renverse accidentellement son café parterre et cherche quelqu'un pour nettoyer les dégâts.
3. Antoine va trouver Lucas, le serveur, qui est dans la cuisine.
4. Lucas demande à Antoine « Vous venez pour payer le café ? »

False Belief 9

(Not used)

1. ~~Julie vient d'emménager dans une nouvelle maison sans rideaux.~~
2. ~~Juste après le déménagement, Julie va faire du shopping, s’achète de nouveaux rideaux et les accroche.~~
3. ~~Sa meilleure amie, Lisa, passe voir sa nouvelle maison et lui dit : « Oh, ces vieux rideaux ne vont pas du tout dans la pièce ! Tu devrais en acheter de nouveaux ! »~~
4. ~~Julie demande : « Veux-tu voir le reste de ma maison ? »~~

False Belief 10

1. La mère d’Hélène organise une fête surprise pour l'anniversaire d’Hélène. Elle invite Marine et lui dit : « Ne dis rien à personne, surtout pas à Hélène ! »
2. La veille de la fête, Marine déchire sa nouvelle robe alors qu’elle est en train de jouer avec Hélène.
3. « Oh », dit Marine, « Je voulais la porter à ta fête. »
4. « Quelle fête ? » demande Hélène. « Viens », dit Marine. "Demandons à ma mère si elle peut réparer ma robe ».

TRUE BELIEF STORIES (adapted from Baron-Cohen et al., 1999; translated from Dutch)

True Belief 1

1. Toute la classe participe au concours de poésie. Jeanne voudrait vraiment gagner.
2. Alors que Jeanne est à l’école, les résultats du concours sont annoncés. La gagnante est Marie.
3. Un peu plus tard, à l’école, Jeanne rencontre Marie. Marie lui dit : "Je suis désolée pour ton poème".
4. « Je suis vraiment triste ! » dit Jeanne. "Je comprends", répond Marie.

True Belief 2

1. David s’est inscrit dans une nouvelle école et s'est lié d'amitié avec ses camarades de classe Antoine et Michel.
2. Pendant la pause, Antoine se plaint auprès de Michel et David en disant « Toutes les écoles et tous les professeurs sont épouvantables.»
3. David est dans les parages et dit à Antoine : « Ma mère travaille dans une confiserie. Tu veux des bonbons ? »
4. Antoine dit : « Désolé », je ne me sens pas bien. »

True Belief 3

1. Jean est dans les toilettes de l'école. Nicolas et Antoine sont près des lavabos.
2. Nicolas dit : « Bonjour le nouveau, Jean, tu es à l'intérieur? »
3. Jean sort des toilettes.
4. Antoine dit : « Salut Jean. Tu viens jouer au football avec nous ? »

True Belief 4

1. Avant la visite de son voisin, Caroline aide sa mère à faire une tarte aux fruits.
2. Quand le voisin arrive, Caroline va chercher la tarte à la cuisine.
3. Caroline montre la tarte au voisin et lui dit fièrement : « J'ai fait cette tarte spécialement pour toi".
4. "Mmm", dit le voisin, « Cette tarte a l’air bonne. J’adore les tartes, surtout celles aux fruits ! »

True Belief 5

1. Simon donne une voiture miniature au frère de Robert pour son anniversaire alors que Robert est, lui aussi, présent à la fête.
2. Quelques mois plus tard, Simon et Robert jouent avec la voiture.
3. Alors qu’ils sont en train de jouer, Simon laisse tomber la voiture.
4. « Ne t’inquiète pas », dit Robert, « C’était un accident. »

True Belief 6

1. Julie, qui porte de courts cheveux bruns est en visite chez son oncle Matthieu quand on sonne à la porte.
2. C'est Mme Smet, une voisine.
3. Mme Smet dit : « Bonjour » et met ses lunettes. Elle regarda Julie et dit : « Oh, je ne crois pas avoir déjà rencontré cette petite fille. Comment t’appelles-tu ? »
4. Oncle Matthieu dit : « Cette petite fille s’appelle Julie ».

True Belief 7

1. Avant de commencer le cours, le professeur dit : « Vous connaissez tous Benoît, votre camarade de classe. Il est très gravement malade. ».
2. Les élèves sont tristes et silencieux. Une petite fille prénommée Anne se met à pleurer.
3. "Désolée d'avoir pleuré, je viens d'aller chez le dentiste", dit Anne.
4. Le professeur dit à Anne « Va t’asseoir et continue ton travail. »

True Belief 8

1. Alain commande un coca dans un restaurant.
2. Alain renverse accidentellement son coca parterre et cherche quelqu’un pour nettoyer les dégâts.
3. Alain va voir Guy, le serveur, qui se tient dans le restaurant.
4. Guy demande à Alain : « Je nettoie le coca parterre? »

True Belief 9

(Not used)

1. ~~Michèle vient d'emménager dans sa nouvelle maison et n’a pas de tapis pour sa chambre à coucher.~~
2. ~~Juste après le déménagement, Michèle va faire du shopping avec son amie Samantha, achète un nouveau tapis et le met dans sa chambre.~~
3. ~~Samantha regarde la chambre et dit : « Oh, ton nouveau tapis ne donne pas bien dans la chambre ! Tu dois t’en acheter un autre ! »~~
4. ~~Michèle demande « Et tu aimes le reste de la maison ? »~~

True Belief 10

1. La mère d'Annabelle organise une grande fête pour l'anniversaire d'Annabelle. Elle invite Barbara et lui dit : « S’il te plaît, parles-en à tous les amis d'Annabelle ! »
2. La veille de la fête, Annabelle et Barbara jouent ensemble et Barbara déchire sa nouvelle robe.
3. « Oh » dit Barbara « Je voulais porter cette robe à ta fête. »
4. « C’est peut-être encore possible » dit Annabelle. « Viens », dit Barbara « Demandons à ma mère si elle peut la réparer. »

MECHANICAL (Novel, best translation from Dutch)

Mechanical 1

1. Pierre prend le tournant à grande vitesse avec son vélo.
2. Le pneu du vélo heurte une pomme de pin, qui se met à rouler sous l’effet du choc.
3. La pomme de pin descend la colline de plus en plus vite en direction du ruisseau
4. La pomme de pin est emportée par le courant du ruisseau.

Mechanical 2

1. Le hors-bord avec lequel Eric fait la course sort du virage à grande vitesse et heurte un rocher.
2. Le hors-bord heurte le rocher avec beaucoup de bruit. Un morceau de pierre se détache et est emporté dans les airs.
3. Le morceau de pierre se dirige vers un autre bateau à une vitesse exponentielle.
4. La pierre volante heurte la coque de l’autre bateau et la transperce.

Mechanical 3

1. Un vent violent souffle à travers la fenêtre de la chambre de Michaël.
2. La rafale ferme la porte avec violence.
3. Le choc fait vibrer un vase en verre sur l'armoire juste à côté de la porte.
4. Le vase tombe de l'armoire et éclate en mille morceaux.

Mechanical 4

1. Une rafale de vent ferme la porte de l’armoire avec violence.
2. Sous le choc, une balle noire qui se trouve sur l’armoire se met à rouler.
3. La balle noire tombe de l’armoire sur le sol en direction de la table.
4. La balle noire roule sous la table et s’arrête contre le pied de table le plus éloigné.

Mechanical 5

1. Le soleil vient d’apparaître à l’horizon.
2. Le soleil est haut dans le ciel.
3. Le soleil commence à descendre derrière les dunes.
4. Le soleil a totalement disparu derrière l’horizon.

Mechanical 6

(Not used)

Mechanical 7

1. Il n’y avait pas de nuages dans le ciel.
2. Les nuages ont commencé à s’accumuler en cours d’après-midi.
3. Plus tard, il a commencé à pleuvoir.
4. Après plusieurs jours de pluie, le ruisseau a débordé.

Mechanical 8

1. Le volcan est inactif depuis des années.
2. Le volcan commence à cracher de la lave.
3. La lave en fusion commence à dévaler les flancs de la montagne.
4. La lave bouillante se déverse dans la mer.

Mechanical 9

1. Toutes les voitures prennent la route tôt le matin.
2. La circulation sur l'autoroute est fluide, mais une dizaine de minutes plus tard, cela commence à ralentir.
3. Les voitures commencent à rouler au pas et finissent par s’immobiliser dans l’embouteillage.
4. L’ embouteillage se résorbe lentement et la circulation reprend enfin.

Mechanical 10

1. Au pôle Sud, une petite fissure apparaît soudain dans la glace.
2. Après quelques jours, la fissure s’étend.
3. La masse de glace se rompt et s’éloigne du continent.
4. La masse de glace dérive sur la mer.

SOCIAL SCRIPTS (Novel, best translation from Dutch)

Social Script 1

(Not used)

1. ~~Thomas entre dans le supermarché et prend un panier à l'entrée.~~
2. ~~Thomas prend des pâtes, de la sauce tomate et du fromage et les dépose dans le panier.~~
3. ~~À la caisse, Tomas met ses courses dans son sac et paie avec sa carte bancaire.~~
4. ~~En quittant le magasin, Thomas laisse le panier dans le magasin.~~

Social Script 2.

1. Sylvie se dirige vers les cabines d'essayage et attend son tour.
2. Quand une des cabines se libère, Sylvie dit à la vendeuse combien de pièces elle souhaite essayer.
3. Sylvie essaie deux tee-shirts et une jupe. Elle décide d'acheter la jupe.
4. Sylvie quitte la cabine d'essayage et laisse les deux tee-shirts à la vendeuse.

Social Script 3

1. Olivier sort son téléphone portable de sa poche et l’active.
2. Olivier forme le numéro d’un ami auquel il veut poser une question.
3. Comme son ami ne répond pas, Olivier lui laisse un message lui demandant de le rappeler.
4. Olivier raccroche et remet son portable dans sa poche.

Social Script 4

1. Yasmine achète une carte d'anniversaire pour son amie à la librairie.
2. Yasmine écrit un gentil message et met la carte dans une enveloppe.
3. Yasmine ferme l'enveloppe, y inscrit l’adresse et y colle un timbre-poste.
4. Yasmine se rend à la boîte aux lettres et y glisse l’enveloppe.

Social Script 5.

1. Catherine cherche des meubles sur un site internet pour aménager son appartement.
2. Catherine fait son choix, sélectionne les meubles et clique pour payer.
3. Catherine choisit le mode de paiement et introduit les données demandées.
4. Le site Web signale que l’achat de Catherine a bien été effectué.

Social Script 6.

1. Christian entre dans la bibliothèque et cherche dans les rayons pour trouver le livre qu’il souhaite lire.
2. Quand Christian trouve le livre, il l'emmène au comptoir de prêt.
3. Au comptoir de prêt, Christian scanne son badge et le livre.
4. Christian ramène le livre à la maison.

Social Script 7.

1. Christophe met une casserole remplie d’eau sur le feu.
2. Quand l'eau commence à bouillir, Christophe verse les pâtes dans la casserole.
3. Christophe attend quelques minutes jusqu’à ce que les pâtes soient cuites.
4. Christophe égoutte les pâtes et les mange.

Social Script 8

1. Benoît entre dans le cinéma et regarde l’offre de films.
2. Benoît achète un ticket pour le film qu’il souhaite voir.
3. Avant d’entrer dans la salle, Benoît achète des chips et une boisson.
4. Benoît s’installe dans un fauteuil et regarde le film.

Social Script 9.

1. Stéphane consulte l'horaire du bus. Quand le bus est sur le point d'arriver, il se dirige vers l'arrêt.
2. Quand le bus arrive, Stéphane monte et scanne sa carte de bus.
3. Quand le bus s’approche de son arrêt, Stéphane appuie sur la sonnette.
4. Stéphane sort du bus et se rend à sa destination.

Social Script 10

1. Jean est assis dans un restaurant et regarde la carte.
2. Jean appelle le garçon et commande un plat et une boisson.
3. Lorsqu’il a terminé son repas, Jean appelle le garçon et demande l’addition
4. Jean paie son plat et sa boisson et donne un petit pourboire.

**Story Sequencing test (IT)**

Istruzioni: ordinare cronologicamente le frasi dei racconti.

PRACTICE STORIES

Practice 1

1. Prendendo la chiave dalla tasca della giacca, Silvia non si accorge che le cade un pezzetto di carta.
2. Non appena tocca terra, il foglietto viene portato via da una folata di vento.
3. Il foglietto svolazza per alcuni metri, per poi tornare lentamente a terra.
4. Il foglietto si ferma nel canale di scolo.

Practice 2

1. Raffaele mette il guinzaglio al cane ed esce di casa.
2. Raffaele passeggia con il cane sul marciapiede in direzione del parco.
3. All'interno del parco Raffaele lascia il guinzaglio allungato.
4. Raffaele richiama a sé il cane con il guinzaglio e si dirige verso casa a piedi.

FALSE BELIEF STORIES (adapted from Baron-Cohen et al., 1999; translated from Dutch)

 False Belief 1

1. Tutta la classe partecipò alla gara di racconti. Emma avrebbe tanto voluto vincere.
2. Mentre Emma era assente da scuola, furono annunciati i risultati della gara di racconti: aveva vinto Alice.
3. Dopo il rientro di Emma a scuola, Alice la vide e le disse: "Mi spiace per il tuo racconto."
4. "Che intendi?" chiese Emma. "No, niente" rispose Alice.

False Belief 2

1. Roberto era da poco in una nuova scuola e aveva fatto amicizia con alcuni compagni di classe: Clara e Andrea.
2. Durante la ricreazione, Clara si lagnava solo con Andrea dicendo: "Tutte le scuole e tutti gli insegnanti sono orribili. "
3. Roberto arrivò poco dopo e chiese a Clara: "Mia madre è un'insegnante di questa scuola. Vuoi venire con noi a casa a giocare a Monopoly?"
4. Clara rispose: "Scusa, non mi sento bene."

False Belief 3

1. Mike era chiuso in uno dei bagni della scuola. Giovanni e Piero erano vicini ai lavandini e non si erano di accorti di Mike.
2. Mike sentì Giovanni dire agli altri: "Avete presente il ragazzo nuovo? Si chiama Mike. Non vi sembra strano?"
3. Mike uscì dal bagno.
4. Piero disse: "Ehi, ciao Mike, vieni con noi a giocare a calcio?"

False Belief 4

1. Per la visita dello zio Tommaso, Camilla aiutò sua madre a fare una torta speciale con delle mele invisibile all'interno dell'impasto.
2. Dopo l’arrivo dello zio Tommaso, Camilla andò a prendere la torta in cucina.
3. Camilla fece vedere la torta allo zio Tommaso e disse fiera: "Ho fatto questa torta per te.”
4. "Mmm", disse lo zio Tommaso, "questa torta ha un aspetto delizioso. Adoro le torte, tranne le torte di mele, ovviamente!"

False Belief 5

1. Jacopo regalò un aeroplanino giocattolo al fratello di Riccardo per il suo compleanno, mentre Riccardo era in vacanza.
2. Alcuni mesi dopo, Jacopo e Riccardo stavano giocando con l'aeroplanino.
3. Mentre Jacopo e Riccardo giocavano, Jacopo per sbaglio fece cadere l'aeroplanino.
4. "Non preoccuparti", disse Riccardo a Jacopo, "Non mi è mai piaciuto quell'aeroplanino. L'aveva regalato qualcuno a mio fratello per il suo compleanno."

False Belief 6

1. Sara, che ha i capelli corti e biondi, era a casa di sua zia Chiara quando qualcuno suonò alla porta.
2. Era Maria, una vicina.
3. Maria disse: "Ciao" e si tolse gli occhiali. Guardò Sara e disse: "Ma chi è questo ragazzino? Non mi sembra di conoscerlo. Come ti chiami?"
4. La zia Chiara rispose: “Questa signorina è Sara."

False Belief 7

1. All'inizio della lezione l'insegnante disse: "Uno dei ragazzi della nostra classe, Simone, è gravemente malato".
2. Gli alunni diventarono tutti molto tristi e rimasero in silenzio, quando una ragazzina, Rebecca, entrò in classe in ritardo.
3. "Ve l'ho raccontata la mia ultima barzelletta sui malati?" chiese Rebecca.
4. L'insegnante non rise e disse a Rebecca: "Vai a sederti e comincia a lavorare."

False Belief 8

1. Valerio stava facendo colazione in un ristorante.
2. Valerio rovesciò per sbaglio il caffè per terra e si mise a cercare qualcuno che pulisse quel disastro.
3. Valerio andò da Giorgio, il cameriere, che era in cucina.
4. Giorgio chiese a Valerio: "Desidera pagare il caffè?”

False Belief 9

(Not used)

1. ~~Giulia aveva appena traslocato in una nuova casa senza tende.~~
2. ~~Subito dopo il trasloco, Giulia andò a fare shopping, comprò delle tende nuove e le appese.~~
3. ~~La sua migliore amica Lisa andò a trovarla e disse: "Queste vecchie tende non si adattano all'arredamento della stanza! Ne devi comprare di nuove!"~~
4. ~~Giulia chiese: "Vuoi vedere il resto della casa?"~~

False Belief 10

1. La mamma di Elena aveva organizzato una festa a sorpresa per il compleanno di Elena. Aveva invitato Nicoletta dicendole: "Non dirlo a nessuno, soprattutto non dirlo a Elena!"
2. Il giorno prima della festa Nicoletta e Elena stavano giocando insieme quando a Nicoletta si strappò il vestito.
3. "Oh no!" disse Nicoletta, "volevo metterlo alla tua festa."
4. "Che festa?" chiese Elena. "Dai," disse Nicoletta, "chiediamo a mia mamma se lo può riparare."

TRUE BELIEF STORIES (adapted from Baron-Cohen et al., 1999; translated from Dutch)

True Belief 1

1. Tutta la classe partecipò alla gara di poesia. Gianna avrebbe tanto voluto vincere.
2. Mentre Gianna era scuola, furono annunciati i risultati della gara di poesia: aveva vinto Maria.
3. Più tardi a scuola Gianna incontrò Maria. Maria disse: "Mi spiace per la tua poesia. Come ti senti?".
4. "Mi sento malissimo! " disse Gianna. "Lo capisco" disse Maria.

True Belief 2

1. Davide era da poco in una nuova scuola e aveva fatto amicizia con alcuni suoi compagni di classe: Gianfranco e Michele.
2. Durante la ricreazione Gianfranco si lagnava con Michele e Davide dicendo: "Tutte le scuole e gli insegnanti sono orribili. "
3. Davide era presente e chiese a Gianfranco: "Mia madre lavora in un negozio di dolciumi, vuoi delle caramelle?"
4. Gianfranco rispose: "Scusa, non mi sento bene."

True Belief 3

1. Giacomo era chiuso in uno dei bagni della scuola. Samuele ed Ezio erano vicini ai lavandini.
2. Samuele disse "Ciao Giacomo, compagno nuovo, sei lì dentro?"
3. Giacomo uscì dal bagno.
4. Ezio disse: "Ehi, ciao Giacomo. Vieni con noi a giocare a calcio?"

True Belief 4

1. Per la visita del suo vicino di casa, Caterina aiutò la mamma a fare una torta speciale con della frutta sopra.
2. Dopo l’arrivo del vicino, Caterina andò a prendere la torta in cucina.
3. Caterina fece vedere la torta al vicino e disse fiera: "Ho fatto questa torta per te.”
4. "Mmm", disse il vicino, "questa torta ha un aspetto delizioso. Io adoro le torte, soprattutto quelle alla frutta."

True Belief 5

1. Simone regalò al fratello di Roberto un'automobilina giocattolo in occasione della sua festa di compleanno, a cui era presente anche Roberto.
2. Qualche mese dopo Simone e Roberto stavano giocando con la macchinina.
3. Mentre Simone e Roberto giocavano, Simone fece cadere la macchinina.
4. "Non preoccuparti", disse Roberto, "è stato un incidente".

True Belief 6

1. Giulia, che ha i capelli corti e castani, era a casa di suo zio Tony quando qualcuno suonò alla porta.
2. Era la signora Bianchi, una vicina.
3. La signora Bianchi disse: "Ciao" e si mise gli occhiali. Guardò Giulia e disse: "Ma chi è questa ragazzina? Non mi sembra di conoscerla. Come ti chiami?"
4. Lo Zio Tony disse: "Questa signorina è Giulia".

True Belief 7

1. All'inizio della lezione l'insegnante disse alla classe: "Conoscete tutti il nostro compagno Giordano, è molto molto malato".
2. Gli alunni diventarono tutti molto tristi e rimasero in silenzio. Una bambina, Anna, cominciò a piangere.
3. "Scusate se piango, sono appena stata dal dentista", disse Anna.
4. L'insegnante disse ad Anna: "Vai a sederti e continua a lavorare."

True Belief 8

1. Alessandro ordinò una coca-cola in un ristorante.
2. Alessandro rovesciò per sbaglio la coca per terra e si mise a cercare qualcuno che pulisse quel disastro.
3. Alessandro andò da Edoardo, il cameriere, che era lì nel ristorante.
4. Edoardo chiese ad Alessandro: "Vuoi che venga a pulire per terra?"

True Belief 9

(Not used)

1. ~~Michela aveva appena traslocato nella nuova casa e non aveva un tappeto in camera da letto.~~
2. ~~Subito dopo il trasloco, Michela andò a fare shopping con la sua amica Samantha, comprò un tappeto nuovo e lo mise in camera da letto.~~
3. ~~Samantha osservò la stanza e disse: "Oh, ma il tappeto nuovo non sta bene in camera da letto! Ne devi comprare un altro!"~~
4. ~~Michela le chiese: "Il resto della casa ti piace?"~~

True Belief 10

1. La mamma di Annalisa aveva organizzato una grande festa per il compleanno di Annalisa. Aveva invitato Barbara dicendole: "Per favore dillo a tutti gli amici di Annalisa!"
2. Il giorno prima della festa Annalisa e Barbara stavano giocando insieme, quando a Barbara si strappò il vestito nuovo.
3. "Ma no!" disse Barbara, "volevo metterlo alla tua festa.”
4. "Forse è ancora possibile" disse Annalisa. "Dai" disse Barbara "chiediamo a mia mamma se lo può riparare."

MECHANICAL (Novel, best translation from Dutch)

Mechanical 1

1. Pietro prese la curva in bicicletta a tutta velocità.
2. La ruota della bicicletta andò a sbattere contro una pigna, che ruzzolò via per l'urto.
3. La pigna cominciò a rotolare sempre più velocemente lungo la discesa in direzione del ruscello.
4. La pigna si allontanò portata via dalla forte corrente del ruscello.

Mechanical 2

1. Alla curva il motoscafo su cui gareggiava Enrico andò a schiantarsi a tutta velocità contro una roccia.
2. Il motoscafo colpì rumorosamente la roccia e dalla roccia si staccò un pezzo che volò via.
3. Il pezzo di roccia finì con una velocità vertiginosa contro un'altra barca.
4. Il pezzo di roccia volante andò a urtare contro lo scafo dell'altra barca, in cui fece un buco.

Mechanical 3

1. Una violenta folata di vento entrò dalla finestra nella camera di Michele.
2. A causa della raffica di vento la porta si chiuse sbattendo rumorosamente.
3. L'urto fece oscillare un vaso di vetro che si trovava sul comò accanto alla porta.
4. Il vaso cadde dal mobile finendo per terra e frantumandosi in mille piccoli pezzi.

Mechanical 4

1. Il vento fece sbattere violentemente lo sportello del comò che si chiuse.
2. Sul mobile c'era una pallina nera che iniziò a rotolare per via dell'urto.
3. La pallina nera rotolò dal comò per terra in direzione del tavolo.
4. La pallina nera continuò a rotolare sotto il tavolo e si fermò contro la gamba del tavolo più lontana.

Mechanical 5

1. Il sole sorse appena sopra l’orizzonte.
2. Il sole era alto nel cielo.
3. Il sole cominciò a calare dietro le dune.
4. Il sole scese completamente dietro l’orizzonte.

Mechanical 6

(Not used)

Mechanical 7

1. In cielo non si vedeva una nuvola.
2. Nel pomeriggio cominciò lentamente a rannuvolarsi.
3. Più tardi iniziò a piovere.
4. Dopo giorni di pioggia, il ruscello straripò.

Mechanical 8

1. Per anni il vulcano era stato inattivo.
2. Il vulcano cominciò a eruttare lava.
3. La lava bollente scorreva sul fianco della montagna.
4. La lava bollente raggiunse il mare.

Mechanical 9

1. Al mattino presto tutte le automobili erano in strada.
2. In autostrada il traffico scorreva molto veloce, ma una decina di minuti dopo la velocità iniziò a diminuire.
3. Le auto cominciarono ad andare a passo d'uomo fino a quando si fermarono completamente incolonnate.
4. Lentamente la coda si dissolse e la circolazione finalmente riprese.

Mechanical 10

1. Al Polo Sud apparve all'improvviso una piccola spaccatura nella massa di ghiaccio.
2. Dopo un paio di giorni la crepa si ingrandì.
3. La massa di ghiaccio si staccò dalla terraferma.
4. La massa di ghiaccio andò alla deriva nel mare.

SOCIAL SCRIPTS (Novel, best translation from Dutch)

Social Script 1

(Not used)

1. ~~Tommaso entrò nel supermercato e prese un cestino all'ingresso.~~
2. ~~Dagli scaffali Tommaso prese pasta, salsa di pomodoro e formaggio e li mise nel cestino.~~
3. ~~Alla cassa Tommaso inserì tutto quanto nel sacchetto e pagò con il bancomat.~~
4. ~~Prima di uscire dal negozio, Tommaso rimise a posto il cestino.~~

Social Script 2

1. Serena si avvicinò ai camerini e aspettò il suo turno.
2. Quando un camerino si liberò, Serena disse alla commessa quanti capi avrebbe voluto provare.
3. Serena provò due maglie e una gonna. Decise di comprare la gonna.
4. Dopo essere uscita dal camerino, Serena lasciò le due maglie alla commessa.

Social Script 3

1. Walter prese il telefonino dalla tasca e accese l'apparecchio.
2. Walter fece il numero di un compagno a cui voleva chiedere una cosa.
3. Non avendo ricevuto risposta dal suo compagno, Walter lasciò un messaggio in segreteria in cui gli chiedeva di richiamarlo.
4. Walter riagganciò e rimise il telefonino in tasca.

Social Script 4

1. Gemma comprò un biglietto di auguri in cartoleria per il compleanno della sua amica.
2. Gemma scrisse un messaggio carino sul biglietto e lo infilò in una busta.
3. Gemma chiuse la busta, scrisse l'indirizzo e vi incollò sopra un francobollo.
4. Gemma si diresse verso la buca delle lettere e infilò il biglietto nella fessura.

Social Script 5

1. Katia cercava dei mobili su un sito internet per arredare il suo appartamento.
2. Quando Katia trovò quello che cercava, selezionò i mobili e cliccò su pagare.
3. Katia scelse un metodo di pagamento e inserì i dati richiesti.
4. Il sito internet comunicò a Katia che il suo acquisto era andato a buon fine.

Social Script 6

1. Christian entrò in biblioteca e si mise a cercare fra gli scaffali il libro che voleva leggere.
2. Quando Christian trovo il libro, lo prese e lo portò al banco del prestito.
3. Al banco del prestito, Christian passò al lettore ottico la sua tessera della biblioteca e il libro.
4. Christian portò il libro a casa.

Social Script 7

1. Cristiano mise sul fornello una pentola con dell'acqua.
2. Quando l'acqua iniziò a bollire, Cristiano buttò la pasta.
3. Cristiano aspettò alcuni minuti prima che la pasta fosse pronta.
4. Cristiano scolò la pasta e la mangiò.

Social Script 8

1. Bernardo entrò nel cinema e guardò l'offerta dei film del giorno.
2. Bernardo acquistò un biglietto per il film che voleva vedere.
3. Prima di entrare in sala, Bernardo comprò delle patatine e una bibita.
4. Bernardo si sedette su una poltrona e guardò il film.

Social Script 9

1. Stefano guardava gli orari dell'autobus. Quando l'autobus stava per arrivare, si incamminò verso la fermata.
2. All'arrivo dell'autobus, Stefano salì e timbrò il biglietto.
3. Poco prima che l'autobus arrivasse alla sua fermata, Stefano suonò per prenotare la fermata.
4. Stefano scese dall'autobus e raggiunse a piedi la sua destinazione.

Social Script 10

1. Giorgio era seduto a un tavolino in un ristorante e osservava il menù.
2. Giorgio chiamò il cameriere e ordinò qualcosa da mangiare e qualcosa da bere.
3. Finito di mangiare, Giorgio chiamò il cameriere e chiese il conto.
4. Giorgio saldò il conto e lasciò una piccola mancia.
